# IRF1 Maintains Optimal Constitutive Expression of Antiviral Genes and Regulates the Early Antiviral Response

**DOI:** 10.3389/fimmu.2019.01019

**Published:** 2019-05-15

**Authors:** Debasis Panda, Erisa Gjinaj, Mahesh Bachu, Erica Squire, Hilary Novatt, Keiko Ozato, Ronald L. Rabin

**Affiliations:** ^1^Center for Biologics Evaluation and Research, US Food and Drug Administration, Silver Spring, MD, United States; ^2^Eunice Kennedy Shriver National Institute of Child Health and Human Development, Bethesda, MD, United States

**Keywords:** antiviral state, transcription factor, interferon independent, epigenetic regulation, basal defense response

## Abstract

Viral defense at mucosal sites depends on interferons (IFN) and IFN stimulated genes (ISGs), either of which may be constitutively expressed to maintain an “antiviral state” (AVS). However, the mechanisms that govern the AVS are poorly defined. Using a BEAS-2B respiratory epithelial cell line deficient in IRF1, we demonstrate higher susceptibility to infection with vesicular stomatitis virus (VSV) and influenza virus. IRF1-mediated restriction of VSV is IFN-independent, as blockade of types I and III IFNs and JAK-STAT signaling before infection did not affect VSV infection of either parent or IRF1 KO cells. Transcriptome analysis revealed that IRF1 regulates constitutive expression of ~300 genes, including antiviral ISGs: *OAS2, BST2*, and *RNASEL* and knockdown of any of these IRF1-dependent genes increased VSV infection. Additionally, IRF1 enhances rapid expression of IFNβ and IFNλ after stimulation with poly I:C and also regulates ISG expression. Mechanistically, IRF1 enhances recruitment of BRD4 to promotor-enhancer regions of ISGs for rapid expression and maintains levels of histone H3K4me1 for optimal constitutive expression. Finally, IRF1 also regulates constitutive expression of TLR2 and TLR3 and promotes signaling through these pattern recognition receptors (PRR). These data reveal multiple roles for IRF1 toward effective anti-viral responses by maintaining IFN-independent constitutive expression of anti-viral ISGs and supporting early IFN-dependent responses to PRR stimulation.

## Introduction

Airway epithelial cells are targets for viral replication and provide the first line of defense against virus entry and infection. The interaction between viral pathogens and epithelial cells often determines the outcome of viral infection, either directly or by modulating the subsequent adaptive immune response. The first step in viral defense is recognition of viral nucleic acids by pattern recognition receptors (PRR) that activates the transcription factors IRF3 and NFκB to induce expression of IFNβ and IFNλ, along with a variety of anti-viral and immunomodulatory interferon-stimulated genes (ISG) that directly or indirectly suppress viral infection (1, 2). Since viral pathogens express proteins to block IFN and ISG functions, innate antiviral immunity is only effective when rapidly implemented. To give the host a head-start, cells acquire an “antiviral state” by expressing ISGs constitutively (i.e., independent of IFNs) or in response to constitutive expression of IFNβ (3–5). IFN-independent constitutive expression of ISGs is appealing because the AVS is acquired without the detrimental bystander effects associated with aberrant IFN expression (6). While the benefits of an IFN-independent AVS are known, molecular mechanisms that regulate it remain undefined.

Interferon regulatory factor-1 (IRF1) is a transcription factor whose expression in respiratory epithelial cells in response to IFNβ is rapid and robust–peaking at 2 h (7). In support of an important role for IRF1 in viral defense, ectopic expression of IRF1 protects otherwise susceptible cells against a diverse range of RNA viruses (8), and IRF1 deficient mice are more susceptible to viral infections (9–13). How IRF1 participates in antiviral defense is unclear (12), in part because its effects are limited to specific tissue sites and may differ according to the viral pathogen. For example, although IRF1 deficient mice succumb to EMCV infection faster than wild type mice, wild type and IRF1 KO mouse embryonic fibroblasts (MEFs) are equally susceptible to EMCV, and neither IRF1 KO mice nor MEFs are susceptible to VSV (12). Adding to the mechanistic complexity is that IRF1 may mediate IFN-dependent or independent responses, by regulating temporal-spatial ISG expression in response to IFNs or enhancing expression of antiviral genes induced by PRR stimulation, respectively. Here we reveal a common epigenetic mechanism of action for IFN-dependent and independent roles for IRF1 using an IRF1-deficient bronchial epithelial cell line.

## Results

### IRF1 Deficiency Enhances Vesicular Stomatitis Virus (VSV) Infection

To explore the role of IRF1 in local immunity, we first demonstrated that BEAS-2B bronchial epithelial cells constitutively express IRF1, and that IFNβ enhances and induces translocation of IRF1 to the nucleus (Supplementary Figure 1A). We then used CRISPR-Cas9 technology to create IRF1 deficient BEAS2B cell lines, and confirmed IRF1 deficiency, both constitutive and 3h post-stimulation with IFNβ, by western blot (Supplementary Figure 1B).

**Figure 1:**
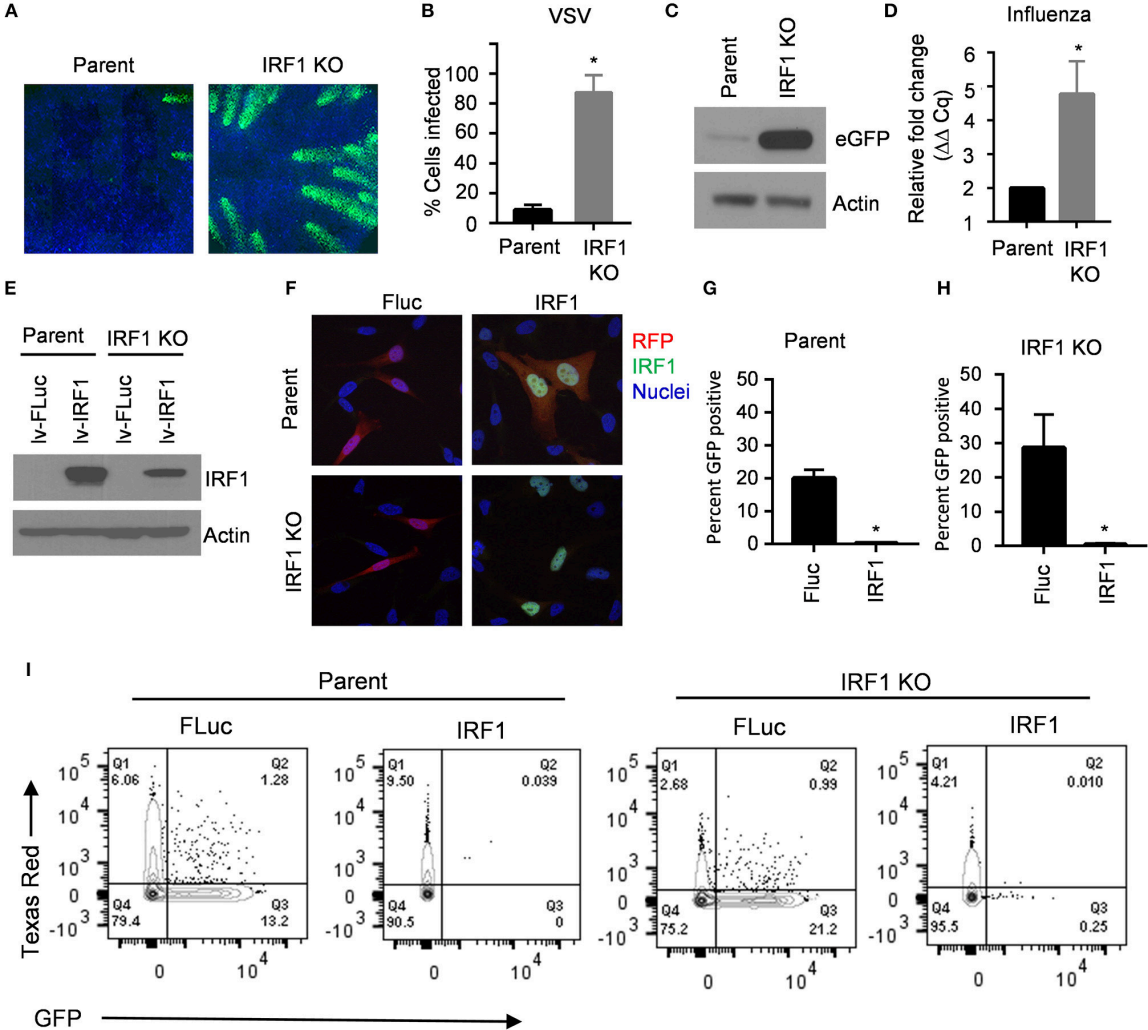
IRF1 is necessary for restriction of VSV and influenza virus in BEAS-2B respiratory epithelial cells. **(A–C)** Parent and IRF1 KO BEAS-2B cells were infected with VSV at 0.01 MOI for 20 h and VSV-encoded GFP expression was examined by imaging **(A)**, flow cytometry **(B)**, and immunoblotting **(C)**. The images shown in panel A are representative of three independent experiments. **(B)** Flow cytometry results of the percent cells infected (GFP+) in three experiments is shown. Mean ±SD is plotted on the graph. **(C)** Immunoblotting for viral encoded GFP is shown. **(D)** Parent and IRF1 KO cells were infected with PR8 influenza virus and influenza matrix gene expression was examined by RT-qPCR. Relative fold change (2^−ΔΔ*Cq*^) is shown. Data represent mean ±SD from three independent experiments. **(E)** Parent or IRF1 KO BEAS-2B cells were transduced with bicistronic lentiviruses encoded for tagRFP and either firefly luciferase (FLuc) or IRF1, and cell lysates were immunoblotted to confirm IRF1 expression. **(F)** Parent and IRF1 KO cells were transduced with the lentiviruses expressing tagRFP and Fluc or IRF1, and IRF1 localization was examined by immunofluorescence microscopy. Representative images are shown. **(G–I)** Parent and IRF1 KO BEAS-2B cells were transduced with lentiviruses, infected with VSV at 0.01 MOI for 20 h, and harvested for flow cytometry. Data shown in **(G)** and **(H)** are mean ± SD from three independent experiments. Representative plots are shown in **(I)**. ^*^Statistically significant.

To characterize the role of IRF1 in antiviral defense, we used a GFP-expressing recombinant VSV (VSV-GFP), a pathogen that is highly sensitive to types I and III IFNs and readily infects respiratory epithelial cells (14). Figures 1A–C shows that compared to parent cells, IRF KO BEAS-2B cells were highly infected with VSV-GFP. IRF1 KO cells were also more susceptible to infection with multiple strains of influenza viruses (Figure 1D and Supplementary Figures 2A–D). A second IRF1 KO BEAS-2B cell line also showed increased susceptibility to VSV infection (Supplementary Figure 2E). To confirm that increased infectivity was due to deletion of IRF1, we transduced cells with a bicistronic lentivirus that expresses tagRFP and IRF1 or as a control, tagRFP and firefly luciferase (lv-IRF1 and lv-Fluc, respectively), (8). In both parent and IRF KO cells, lv-IRF1, but not lv-Fluc induced expression of high levels of IRF1 that had translocated to the nucleus (Figures 1E,F). In addition, after lv-IRF1-induced over-expression of IRF1, both parent and IRF1 KO BEAS-2B cells were completely protected against VSV-GFP (Figures 1G,H). Protection can be achieved either in cis, in which all cells overexpress IRF1, or trans, in which IRF1 expression by a subset of cells induces an AVS throughout the culture. To determine which of these was operative in VSV infection, we measured expression of tagRFP and GFP by flow cytometry. As shown in Figure 1I, transfection with lv-IRF1 induced expression of RFP in only ~4% of the IRF1 KO and overexpression of IRF1 in ~9% of the parent cells, but in each case the RFP^−^ (i.e., without IRF1 overexpression) cells were also protected from VSV-GFP infection, suggesting a strong trans effect.

### IRF1 Is Required for Optimal Early Activation of IRF3 and Expression of Types I and III IFNs

**Figure 2:**
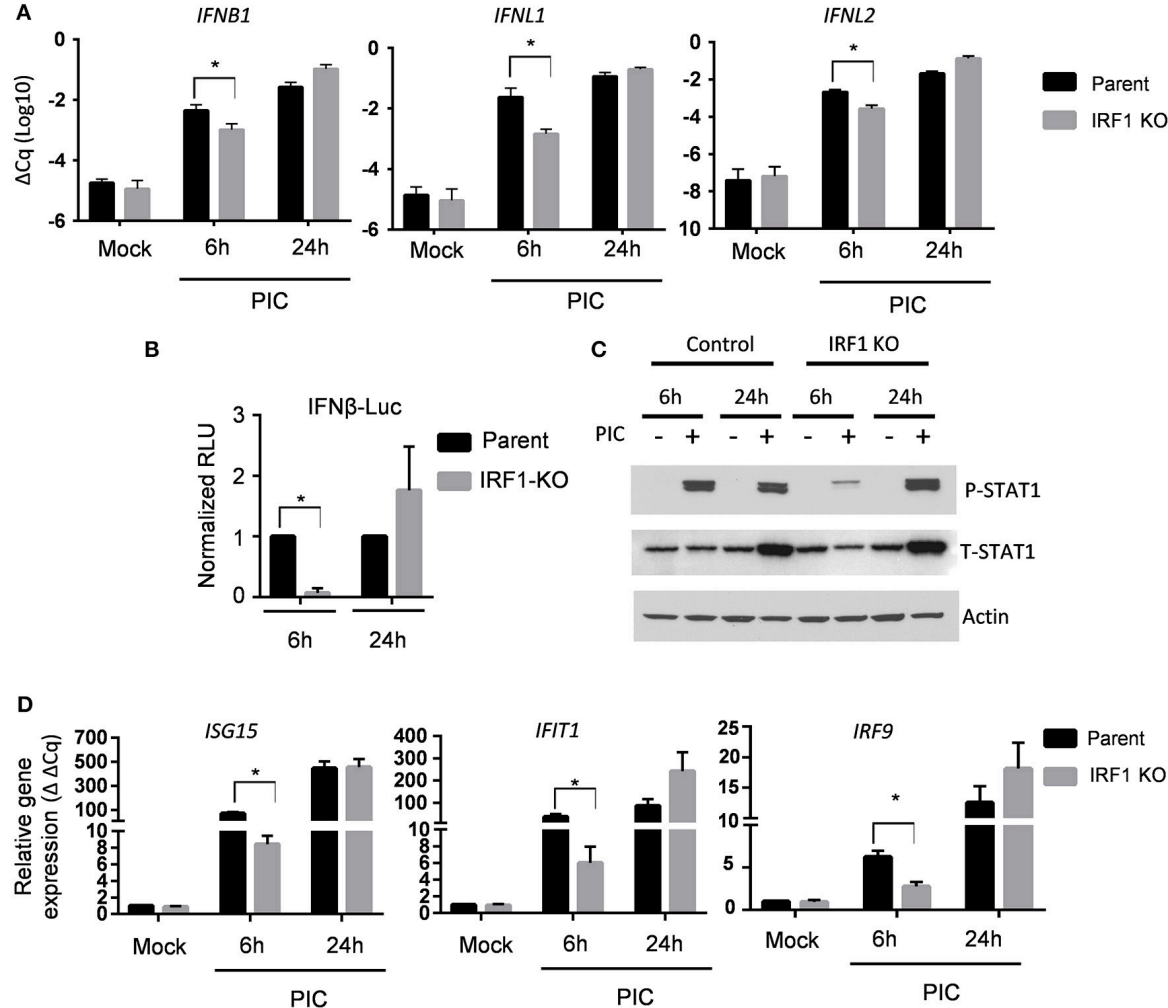
IRF1 is required for early expression of types I and III IFNs and ISG expression. **(A)** Parent BEAS-2B and IRF1 KO cells were transfected with poly I:C, and expression of IFNβ, IFNλ1, or IFNλ2 transcripts were examined by RT-qPCR at 6 h or 24 h after poly I:C transfection. Data represent mean ± SEM from four independent experiments. **(B)** Parent BEAS-2B and IRF1 cells were transfected with a plasmid expressing firefly luciferase under the control of the IFNβ promoter or a plasmid constitutively expressing Renilla luciferase. Cells were then transfected with poly I:C and luciferase expression was examined at 6 h or 24 h afterwards. Firefly luciferase expression was normalized to Renilla luciferase expression and expressed as relative light units (RLU). Data shown are mean ± SD from three independent experiments. **(C)** IRF1 KO and parent cells were transfected with poly I:C, and cell lysates were immunoblotted for STAT1 phosphorylation (Y701). **(D)** Experimental protocol is same as A except that ISG expression was measured by RT-qPCR. Relative gene expression (2^−ΔΔ*Cq*^) is shown. Data represent mean ± SD from three independent experiments. ^*^Statistically significant.

Protection of cells from VSV in trans suggests that IRF1 regulates expression of type I and III IFN. To explore this possibility, we transfected BEAS-2B parent and IRF1 KO cells with poly I:C to activate cytoplasmic PRRs, and measured expression of *IFNB1, IFNL1*, and *IFNL2* by RT-qPCR at 6 h and 24 h. Figure 2A shows that IRF1 KO cells expressed lower levels of these IFN transcripts than parent BEAS-2B cells only at 6 h. We confirmed this selective early effect on IFN expression with a luciferase reporter under the control of the IFNβ promoter (Figure 2B). Consistently, phosphorylation of STAT1 (Y-701) and ISG expression were also decreased in the IRF1 KO cells at 6 h, but not at 24 h after poly I:C transfection (Figures 2C,D).

**Figure 3:**
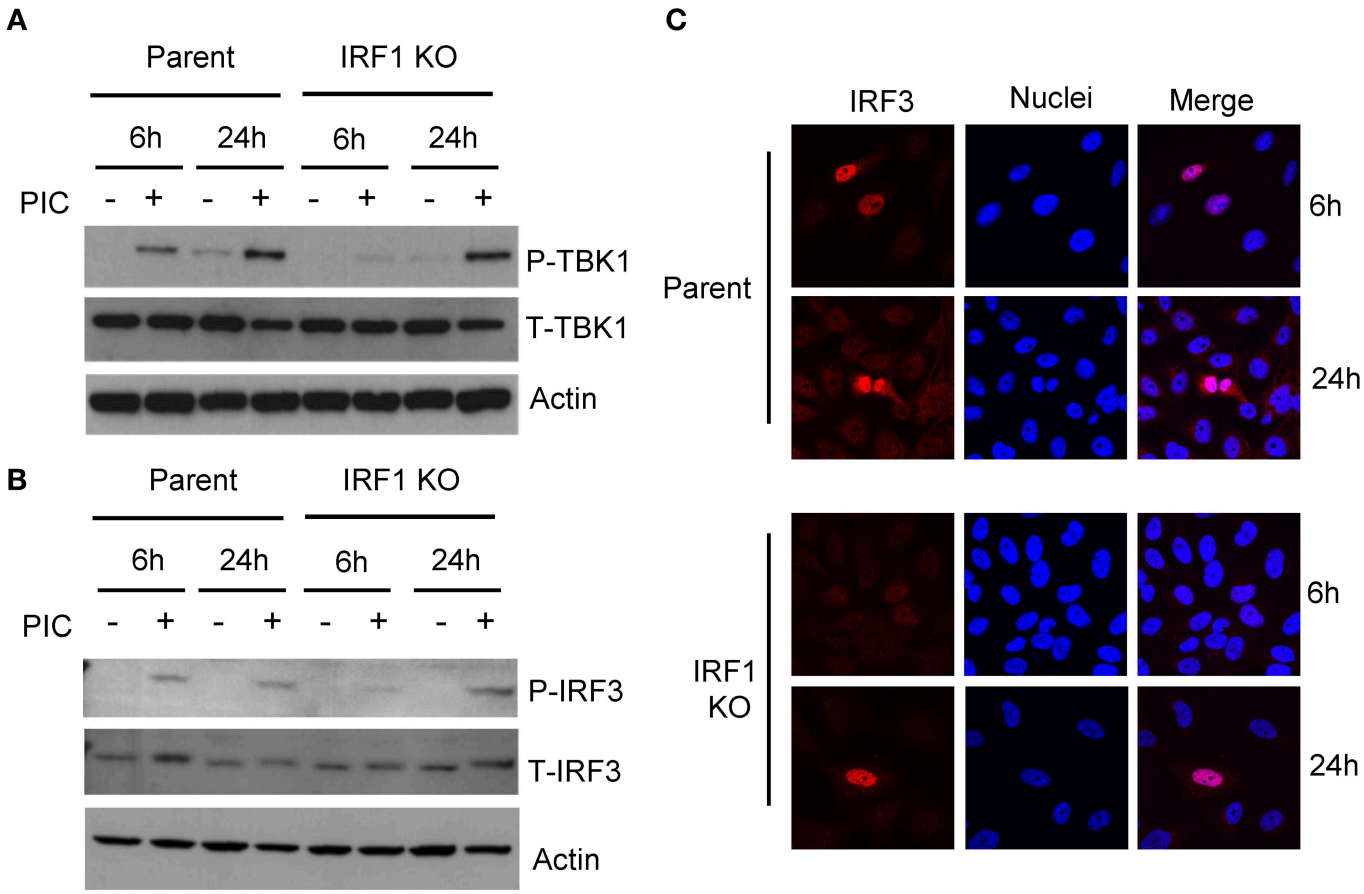
IRF1 is required for optimal early activation of TBK1 and IRF3. Parent BEAS-2B and IRF1 KO cells were transfected with poly I:C and were harvested to measure activation of TBK1, with anti-pTBK1 S172 antibody **(A)** and IRF3 with anti-pIRF3 Y396 antibody **(B)** at 6 h and 24 h by immunoblot. **(C)** IRF1 KO and parent cells were transfected with poly I:C and cells were fixed and immunostained for IRF3. Representative confocal microscopic images are shown.

Since the expression of IFNs and ISGs is IRF3-dependent, we asked whether diminished expression of IFN transcripts and ISGs in IRF1 KO cells is due to diminished IRF3 activation. Figure 3 shows that after transfection of poly I:C, early phosphorylation of TANK binding kinase-1 (TBK1) and its target, IRF3, are decreased in IRF1 KO cells (Figures 3A,B), as is nuclear localization of IRF3 (Figure 3C). No difference in *IRF3* transcript was observed in parent and IRF1 KO cells (Supplementary Figure 3A) Taken together, these data demonstrate that IRF1 enhances early, but not late, IRF3-mediated expression of IFN transcripts, STAT1 activation and ISG expression in respiratory epithelial cells. Thus, IRF1 enhances early, but not late, IFN and ISG expression in part by regulating IRF3 activation.

### IRF1 Does Not Contribute to IFN-Mediated Protection Against VSV

**Figure 4:**
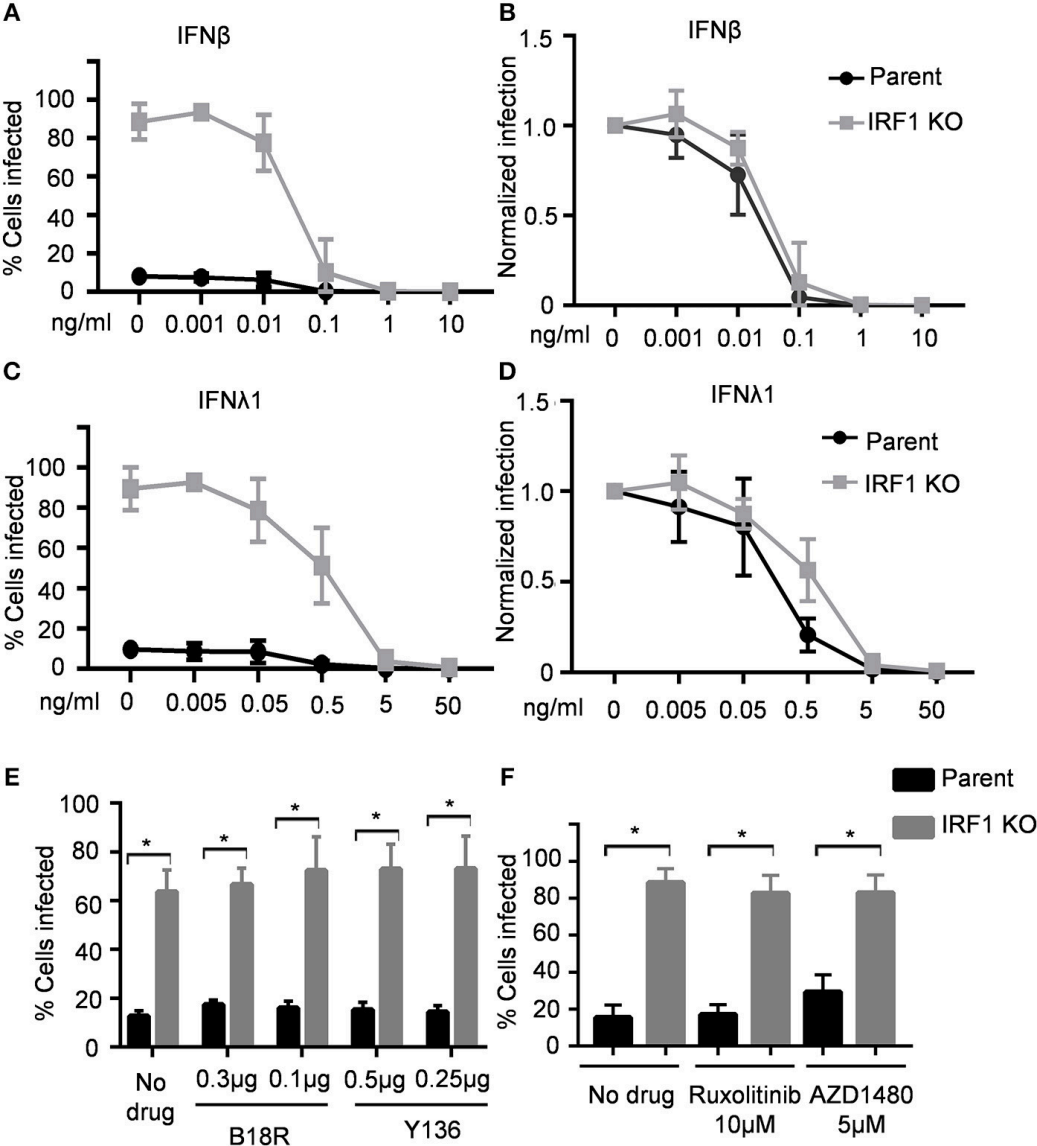
IRF1 is dispensable for IFN-mediated restriction of VSV in BEAS-2B cells. Parent and IRF1 KO cells were pre-treated with IFNβ **(A,B)** or IFNλ1 **(C,D)** for 6 h, then infected with VSV in the presence of IFN, and harvested at 20 hpi for flow cytometry. Data represent mean ± SD from three independent experiments. Percent cells infected is shown in **(A,C)** and normalized infection is shown in **(B,D)**. **(E)** Parent and IRF1 KO BEAS-2B cells were either treated with vehicle control or with B18R or Y136 proteins 24 h before infection. Cells were then infected with VSV at 0.01 MOI in the presence of B18R or Y136 and percent cells infected at 18 hpi was analyzed by flow cytometry. Data represent mean ± SD from three independent experiments. **(F)** Parent and IRF1 KO BEAS-2B cells were either treated with vehicle control or with JAK inhibitors 24 h before infection and then infected with VSV at 0.01 MOI in the presence of the drugs. Percent cells infected at 18 h was analyzed by flow cytometry. Data represent mean ± SD from three independent experiments. ^*^Statistically significant.

Having demonstrated that IRF1 regulates early IRF3 activation, we asked whether IRF1 also directly regulates the JAK/STAT signaling pathway. IFNβ protein was undetectable in VSV infection (not shown) despite induction of types I and III IFN transcripts at low levels (Supplementary Figures 3B,C). Thus, to explore whether IRF1 also directly regulates the JAK/STAT signaling pathway, we asked if exogenous IFNs differentially affects infection of IRF1 KO and parent cells with VSV, a pathogen that is highly sensitive to exogenous type I and III IFNs (14, 15). We therefore pretreated the respiratory epithelial cells with increasing doses of IFNβ and IFNλ1 for 6 h prior to infection with 0.01 MOI of VSV-GFP. As shown in Figure 4A, IFNβ at 0.1 ng/ml protected both parent and IRF1 KO BEAS-2B cells from VSV-GFP infection (Figure 4A). Despite the higher infectivity in untreated IRF1 KO cells, the normalized dose-response curves reveal that the IC50 concentrations for the parent and IRF KO cells are similar (Figure 4B; IC_50_ = 0.019 and 0.028 ng/mL in parent and IRF1 KO cells, respectively). Additionally, IFNλ1 similarly protected parent and IRF1 KO cells from VSV (Figures 4C,D; IC_50_ 0.24 and 0.62 ng/mL in parent and IRF1 KO cells, respectively). Of interest, the lowest IFNβ and IFNλ1 concentrations that completely protected the epithelial cells (0.1 and 5.0 ng/mL, respectively) are also the threshold concentrations that induce detectable ISG expression by these cells (7). Since exogenous IFNs equally protected parent and IRF1 KO BEAS-2B cells, IRF1 function is dispensable for IFN-mediated protection of BEAS-2B cells against VSV.

### Inhibiting Janus Kinase (JAK) Activity and Blocking IFN Receptors Did Not Affect VSV Infection

Since protection from VSV by exogenous IFNs is unaffected by IRF1 deficiency, and IRF1 regulates early IFN expression, we then asked whether IRF1 affects endogenous IFN expression or signaling and thus VSV infection. If so, blockade, either by small molecule inhibitors of JAK/STAT signaling or soluble IFN antagonists would increase VSV expression in parent or IRF1 KO respiratory epithelial cells. We first determined optimal doses of B18R–a high-affinity soluble IFNAR1 analog, and Y136—a soluble receptor for types I and III IFNs, and two JAK inhibitors, ruxolitinib and AZD1480 (Supplementary Figure 4). Figures 4E,F show that neither IFN blockade nor JAK/STAT signaling blockade increased VSV infection of parent or IRF1 KO BEAS-2B cells (Figures 4E,F). Therefore, our results suggest that IRF1-mediated protection of BEAS-2B respiratory epithelial cells from VSV is independent of types I or III IFN.

### IRF1 Regulates Basal Expression of a Subset of Antiviral ISGs

IFN-independent protection may be due to expression of anti-viral genes directly in response to IRF3 activation, or by higher constitutive expression; i.e., a higher AVS. To explore both IFN-dependent and -independent effects of IRF1, we performed RNA-seq of unstimulated and IFN-stimulated parent and IRF1 KO BEAS-2B cells.

**Figure 5:**
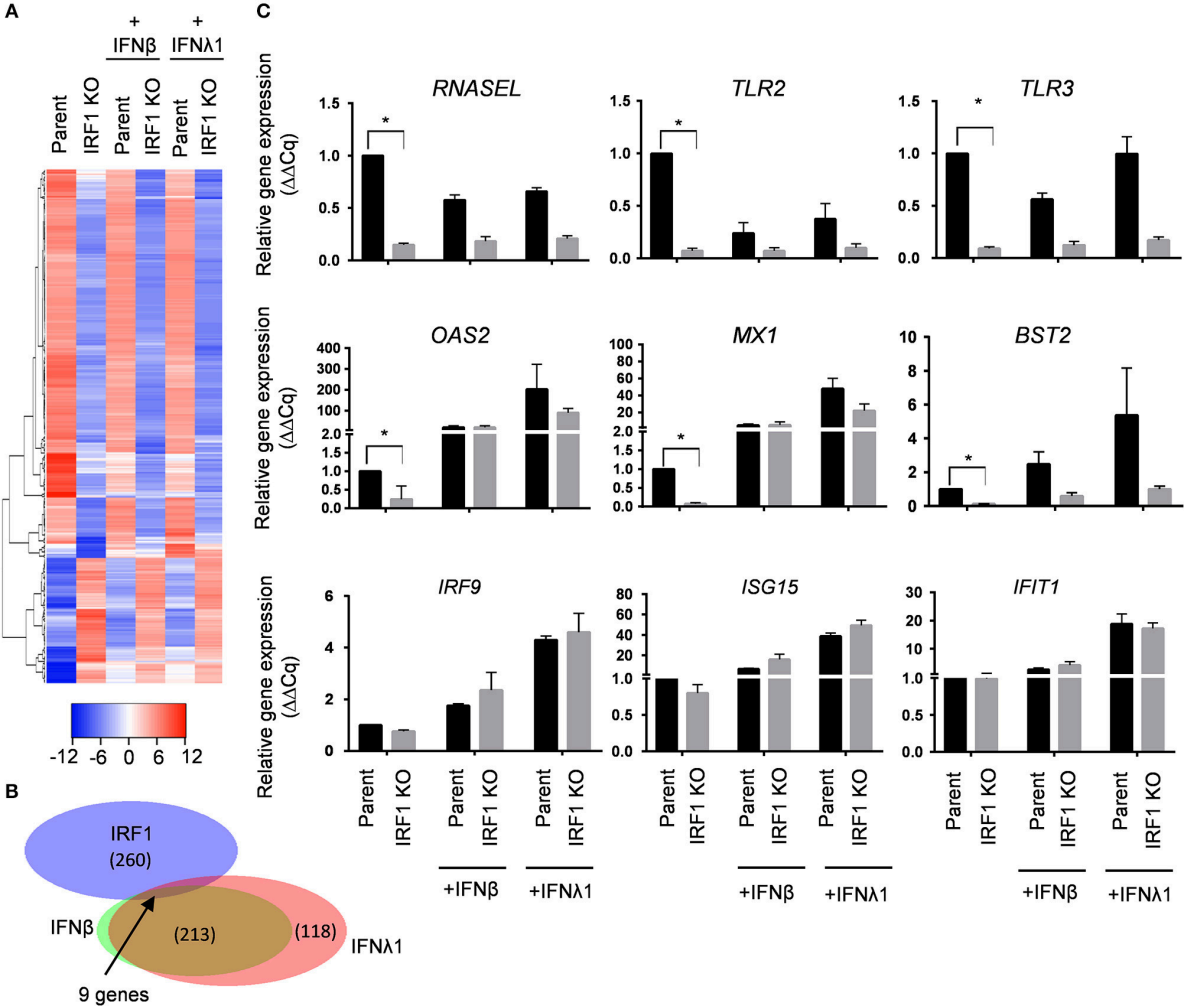
IRF1 regulates basal expression of a subset of ISG in BEAS-2B cells. **(A–C)** Parent and IRF1 KO cells were either treated with IFNβ (0.2 ng/ml) or IFNλ1 (5 ng/ml) for 24 h and gene expression was analyzed by RNA-seq. Heat map **(A)** and Venn diagram **(B)** for IRF1 dependent- and IFN inducible genes are shown. **(C)** Validation of IRF1 dependent, non-ISGs (top row), IRF1 dependent ISGs (middle row) and IRF1-independent ISGs (bottom row) expression by RT-qPCR using comparative C*q* method (2^−ΔΔ*Cq*^). Data represent mean ± SEM from three independent experiments. ^*^Statistically significant.

To define differences in constitutive expression between parent and IRF1 KO cells, we used a stringent 4-fold differences in gene expression as a cutoff and found differential constitutive expression of a total of 340 genes: decreased expression of ~260 genes, and increased expression of ~80 genes by the IRF1 KO cells than parent cells (Figure 5A and Dataset 1). Figure 5B shows that, surprisingly, only nine of these ~340 constitutive differentially expressed genes (DEGs) are also ISGs whose expression by parent cells is also enhanced by IFNs. These nine IRF1 dependent genes are *MX1, BST2, OAS2, DDX60, S100A9, CST6, IFI27, HERC6* and *XAF1*. Figure 5C top, middle and bottom panels, shows validation of constitutive DEGs according to the three representative categories: IRF1 dependent non ISGs (*RNASEL, TLR2, TLR3*); IRF1-dependent ISGs (*OAS2, MX1*, and *BST2*); and to validate similar expression between IRF1 KO and parent cell lines, IRF1-independent ISGs (*IRF9, ISG15*, and *IFIT1*).

To further examine IRF1-dependent genes induced by IFNs, we relaxed the cutoff that defines differential expression to 2-fold and reanalyzed the data (Dataset 1). IFNβ and IFNλ1 stimulated BEAS-2B parent cells to express 268 and 390 genes, respectively, and IRF1 KO cells to express 145 and 183 genes, respectively, above constitutive levels. These results are presented as scatter plots (Supplementary Figures 5A,B,D,E) and volcano plots (Supplementary Figures 5C,F). This less stringent cutoff revealed that IRF1 facilitates expression of approximately half of the IFNβ and IFNλ1 inducible ISGs in these respiratory epithelial cells.

Heat map analysis for the top-30 up- or down-regulated genes provides a closer look at the differential regulations of these genes both at the constitutive level as well as IFN-induced state (Supplementary Figures 6A,B). Ingenuity Pathway Analysis of IRF1 dependent genes revealed enrichment of genes linked to immune signaling pathways such as Toll-like receptor signaling and NF-κB signaling (Supplementary Figures 6C,D). The immune related networks that are affected by IRF1 deficiency are shown in Supplementary Figure 6D. NFκB pathway components are enriched among the IRF1 dependent genes and these genes are involved in a highly interconnected network with cytoskeletal components. Additionally, Ingenuity Pathway Analysis shows TLR3 and TLR2 as members of a highly interconnected network in IFN and NFκB signaling. Of note, the antiviral effector RNASEL is also a member of this network.

### IRF1 Dependent Genes Protect Against VSV Infection

**Figure 6:**
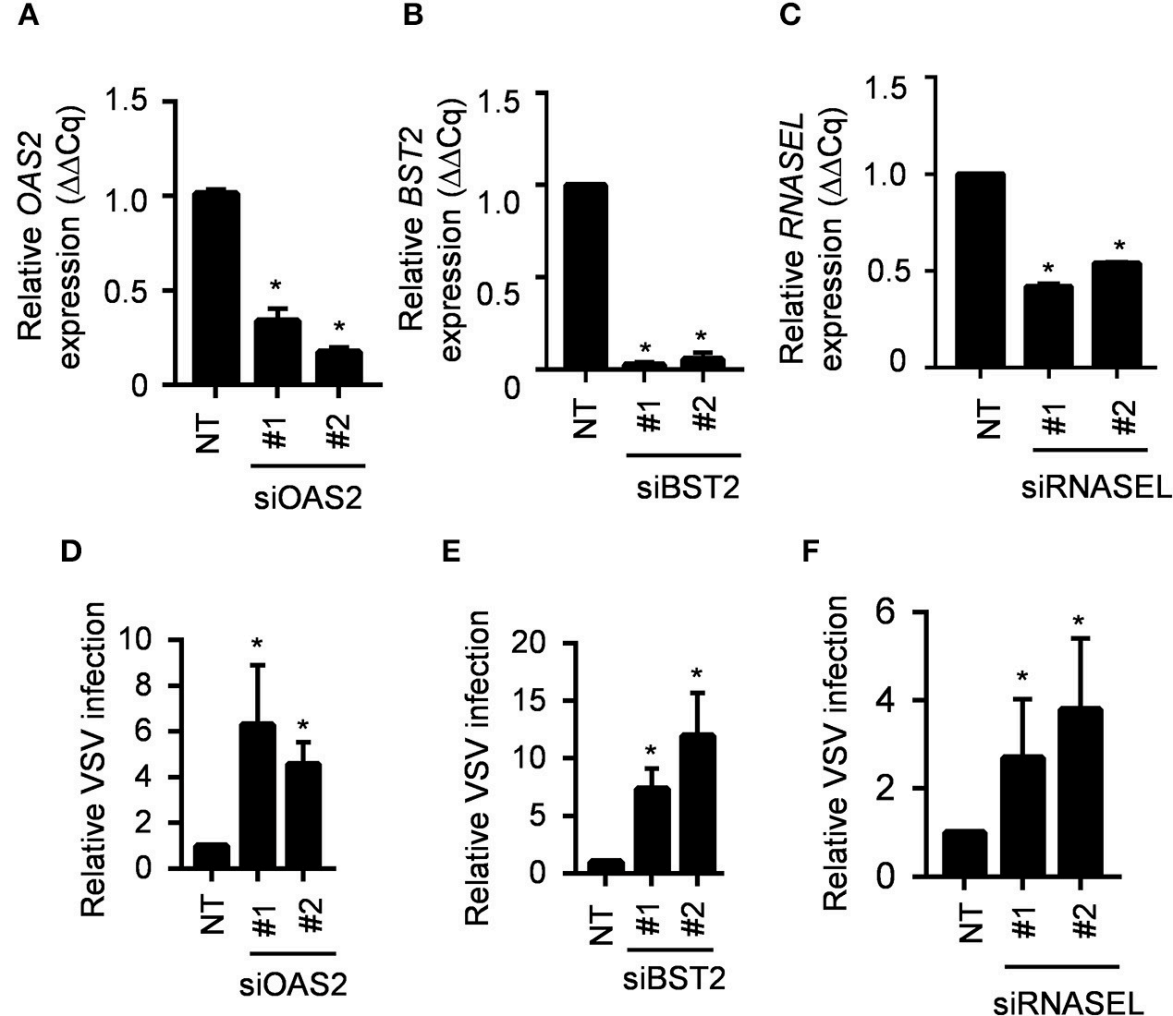
IRF1 dependent genes OAS2, BST2 and RNASEL regulate VSV infection. **(A–C)** Parent BEAS-2B cells were transfected with siRNAs for *OAS2, BST2*, or *RNASEL* and gene expression was analyzed by RT-qPCR using comparative C*q* method (2^−ΔΔ*Cq*^). Data represent mean ± SD from three independent experiments. **(D–F)** Parent BEAS2B cells were transfected with siRNAs for *OAS2, BST2*, or *RNASEL* and 72 h later were infected with VSV (MOI = 0.01). Cells were harvested at 20 hpi for analysis of GFP expression by flow cytometry. Data shown are relative to control siRNA transfected BEAS-2B cells and represent mean ± SD from three independent experiments. ^*^Statistically significant.

Several anti-viral genes are constitutively expressed at lower levels in the IRF1 KO cells. Among these MX1, OAS2 and BST2 are ISGs; RNASEL, however, is not an ISG. BST2 (also known as CD317 or tetherin) prevents release of budding virus from infected cells (16). All three OAS gene products, OAS1-3, catalyze the synthesis of 2',5'-oligoadenylates [2-5As], but may also serve non-redundant functions, and only OAS2 is constitutively expressed by BEAS-2B cells (17). The 2-5As activate RNase L to inhibit viral replication by non-selectively degrading host and viral RNA (18, 19). To further provide a proof of concept that IRF1 dependent genes are indeed antiviral, we explored the importance of these three IRF1-dependent genes by knocking down expression of each in the parent BEAS-2B cells with siRNA before infecting with VSV-GFP. Figure 6 shows that siOAS2, siBST2, and siRNASEL each reduced expression of their target mRNAs and increased VSV-GFP infection compared to the control siRNA treated cells. Therefore, IRF1 maintains the antiviral state of BEAS-2B cells to restrict VSV infection in part by driving constitutive expression of at least three antiviral genes, BST2, OAS2, and RNASEL. Taken together, our selective knockdown of this small subset of the ~300 DEGs demonstrates that IRF1 maintains constitutive expression of antiviral genes, which is one of the many mechanisms by which respiratory epithelial cells may be protected from viral infection.

### IRF1 Deficiency Increases Viral Infection in A549 Respiratory Epithelial Cells

To ensure that our observations are not specific to the BEAS-2B cell line, we developed an IRF1 KO A549 respiratory epithelial cell line using the CRISPR/Cas9 method. Supplementary Figure 7A verifies that IRF1 expression is abolished constitutively or upon IFNβ treatment in the KO cell lines. Similar to the BEAS-2B cells, A549 cells also showed reduced level of STAT-1 phosphorylation in response to poly I:C stimulation (Supplementary Figure 7B), reduced level of constitutive *MX1* expression and increased susceptibility to Influenza infection compared to the parent A549 cells (Supplementary Figures 7C–E).

### IRF1 Mediates Antiviral and Antibacterial PRR Expression

**Figure 7:**
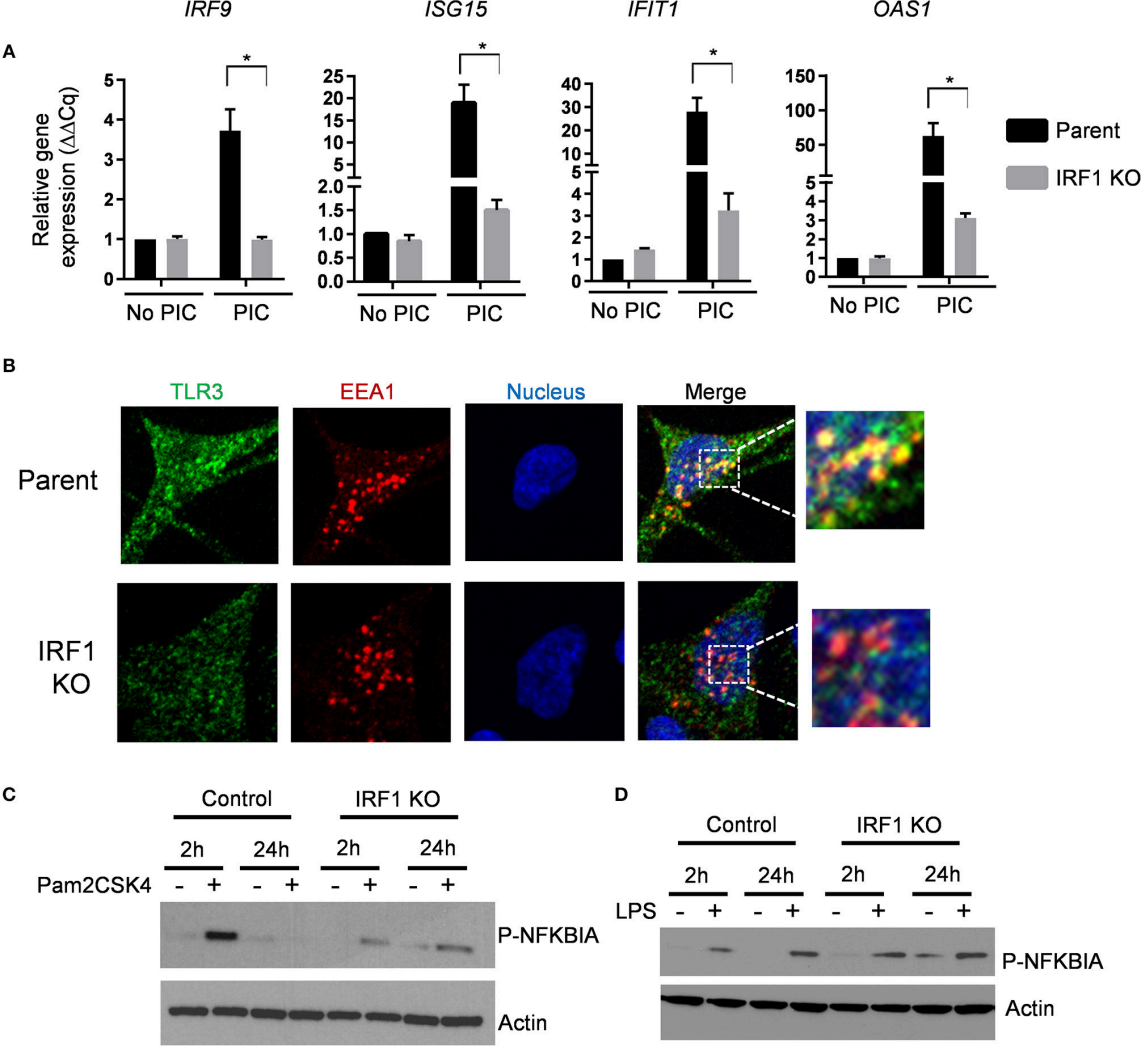
IRF1 regulates TRL2 and TLR3 signaling. **(A)** Parent and IRF1 KO cells were treated with poly I:C (1 μg/mL) for 6 h and IRF1 independent ISG expression was examined by RT-qPCR comparative C*q* method (2^−ΔΔ*Cq*^). Data represent mean ± SEM from three independent experiments. **(B)** Parent and IRF1 KO cells were processed for confocal microscopy to examine localization of TLR3. Antibody against EEA1 was used to examine localization of early endosomes. Images from individual channels for TLR3 (green), EEA1 (red), and nuclei (blue) are shown. Right panels show images with merged channels with magnified view as inset. **(C,D)** Parent and IRF1 KO cells were treated with the respective TLR2 and TLR4 agonists, Pam2CSK4 at 1ng/ml **(C)**, or LPS at 10 ng/ml **(D)** and activation of NF-kB pathway was examined by western blot with anti-phospho-NFKBIA (S32). ^*^Statistically significant.

In addition to antiviral genes, RNA-seq and Ingenuity Pathway Analysis unexpectedly revealed that decreased constitutive expression of *TLR2* and *TLR3* in IRF1 KO cells. Additionally, *S100A9*, which is also reduced in IRF1 KO cells, regulates localization of TLR3 (20). We validated these RNA-seq results and found decreased constitutive expression of TLR3 and TLR2 proteins and verified decreased constitutive mRNA expression of *S100A9* by IRF1 KO cells (Supplementary Figures 8A–C). While transfection with poly I:C activates cytoplasmic RIG-I or MDA-5, simple addition of poly I:C to cells selectively activates TLR3, which resides in endosomes (21). Figure 7A shows that addition of poly I:C for 6h induced robust expression of four ISGs in parent, but not in IRF1 KO cells. Since constitutive expression of each of these four ISGs is unaffected by IRF1 deficiency, constitutive expression and optimal localization of TLR3 are additional IFN-independent mechanisms by which IRF1 mediates antiviral immunity.

To further examine localization of constitutive level of TLR3, we used immunostaining for the early endosomal marker EEA1 and TLR3. Confocal microscopy revealed that TLR3 co-localizes with the EEA1 in the parent cells, but less so in the IRF1 KO cells (Figure 7B). Thus, impaired endosomal localization likely contributes toward impaired TLR3-mediated ISG expression.

To explore functional consequences of reduced TLR2 expression in the IRF1 KO cells, we stimulated parent and IRF1 KO cells with the synthetic TLR2 ligand Pam2CSK4 for 2 h. As shown in Figure 7C, phospho-IκBα, which relieves the inhibitory effect on NF-κB and allows it to localize to the nucleus, was diminished in IRF1 KO cells (Figure 7C) after stimulation of TLR2 with Pam2CSK4, but not after stimulation of TLR4 with LPS. Supplementary Figure 8D shows that expression of *IL1B* was also significantly reduced in Pam2CSK4-stimulated IRF1 KO cells. Thus, in addition to supporting the antiviral state, IRF1 supports anti-bacterial immunity by maintaining constitutive expression of TLR2.

### IRF1 Deficiency Adversely Affects Occupancy of H3K4me1 at the Gene Promoter

We then explored the mechanism by which IRF1 maintains the AVS in respiratory epithelial cells. Post-translational histone modifications facilitate recruitment of transcription factors to promoter sites to initiate or enhance gene transcription. In particular, H3K4me1 promotes the recruitment of specialized transcriptional coupled chromatin readers like Bromodomain-containing protein 4 (BRD4), which recognizes histone tails and yield chromatin landscape amenable to inducible transcription of ISGs (22, 23).

It has been shown that IRF1 regulates gene expression by interacting with both acetylated histones and BRD4 (24, 25). Using chromatin immunoprecipitation assay (ChIP) with anti-IRF1, we first demonstrated occupancy of either constitutive or IFN-inducible IRF1 at the promoter of IRF1-dependent genes. As expected, parent cells only show IRF1 occupancy at the promoter of MX1 and BST2—both are IRF1-dependent genes (Supplementary Figure 9A). By contrast, pull down of promoter region of IRF9 and IFIT1 did not differ between parent and IRF1 KO cells further suggesting minimal occupancy of IRF1 at the promoter of these IRF1-independent genes (Supplementary Figure 9A). Supplementary Figure 9B shows that mRNA expression of these genes correlates with IRF1 occupancy.

**Figure 8:**
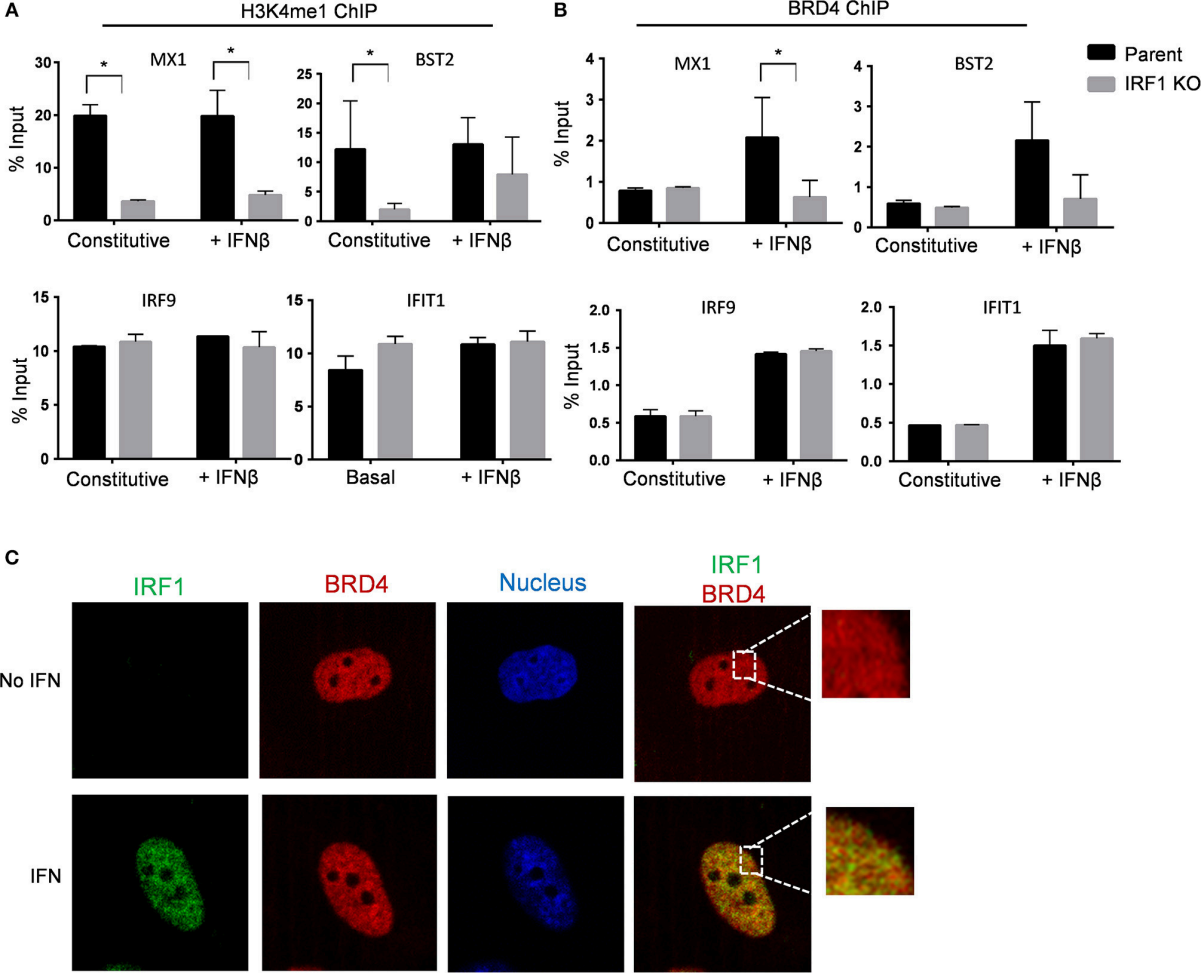
IRF1 dependent H3K4me1 occupancy and recruitment of BRD4 on promoters of IRF1 dependent ISGs. Parent and IRF1-KO cells were treated with IFNβ or left untreated. Chromatin immunoprecipitation was done using H3K4me1 **(A)** and BRD4 **(B)** antibodies. The promoter regions harboring potential IRF1 binding sites of IRF1 dependent genes (MX1 and BST2) and IRF1 independent genes (IRF9 and IFIT1) were analyzed. Percent input (mean ± standard deviation of two biological replicate experiments and three technical replicate PCRs) is expressed as relative enrichment over input (right axis) at steady state and upon IFNβ treatment. **(C)** BEAS-2B cells were either treated with IFNβ or mock treated and then processed for confocal microscopy to examine localization of BRD4 and IRF1. Images from individual channels for IRF1 (green), BRD4 (red), and nuclei (blue) are shown. Right panels show images with merged channels with magnified view as inset. ^*^Statistically significant.

We then used ChIP to compare H3K4me1 occupancy at the enhancer/promoter region of IRF1-dependent genes, MX1 and BST2, to that of IRF1-independent genes, IRF9 and IFIT1 in parent and IRF1 KO cells. As shown in Figure 8A, H3K4me1 occupancy at the promoter/enhancer region of the IRF1-dependent, but not of the IRF1-independent genes, was decreased in IRF1 KO cells either constitutively or after stimulation with IFNβ. Therefore, our results show that IRF1 localizes at the promoter region of IRF1 dependent genes to enhance their constitutive expression.

We then asked whether IRF1 cooperates with BRD4 to enhance the basal antiviral state of BEAS-2B cells by treating cells with a small molecule inhibitor of BRD4, PFI-1, for 24 h before infecting with VSV-GFP. Supplementary Figure 9C shows that inhibition of BRD4 with PFI-1 increased VSV infection of parent and IRF1 KO BEAS-2B cells. While, IFNβ pretreatment reduced VSV infection, treatment with BRD4 significantly relieved this IFNβ-mediated protection and increased VSV infection in parent and IRF1 KO BEAS-2B cells (Supplementary Figure 9D). Consistently, BRD4 inhibition decreased both constitutive and IFN-induced expression of *MX1* and *OAS2* (Supplementary Figures 9E,F). ChIP with anti-BRD4 antibody revealed IFN-dependent increased occupancy of BRD4 at the promoter regions of the IRF1-dependent genes *MX1* and *BST2* only in the parent cells (Figure 8B, top). By contrast, both parent and IRF1 KO cells showed similar level of IFNβ-dependent promoter occupancy of BRD4 for the IRF1-independent genes *IRF9* and *IFIT1* (Figure 8B, bottom). Figure 8B also shows no difference in BRD4 occupancy at the constitutive level in either parent or IRF1 KO cells.

Finally, we confirmed nuclear co-localization of BRD4 and IRF1 by confocal microscopy. IRF1 translocates to the nucleus after IFN stimulation (Figure 8C). By contrast, BRD4 constitutively resides in the nucleus (Figure 8C). Consistent with ChIP analysis, IRF1 colocalized with BRD4 after IFN stimulation, providing further support that IRF1 cooperates with BRD4 to regulate IFN-dependent, but not constitutive gene expression. Taken together, IRF1 enhances IFN-independent constitutive gene expression at least in part by regulating H3K4me1 occupancy and enhances IFN-dependent antiviral protection by modulating BRD4 activity at the promoter regions of IRF1-dependent genes.

## Discussion

Type I interferons are well-known for inducing or enhancing expression of ISGs that block viral entry, replication, or egress. By inducing expression of IRF7, type I interferons also amplify their own expression, as well as that of type III IFNs (IFNλ). Since IRF7 is not constitutively expressed by respiratory epithelial cells, their antiviral defenses may lag behind expression of viral encoded antagonists of antiviral ISGs. Therefore, respiratory epithelial cells must rely on alternative mechanisms, such as enhanced basal expression of ISGs, to block viral replication. To explore the role for IRF1 in antiviral defense, we used BEAS-2B cells as a model because these immortalized human bronchial epithelial cells respond to viral pathogens similarly to that of primary bronchial epithelial cells (26–28). BEAS-2B respiratory epithelial cells constitutively express IRF1, and its expression is rapidly enhanced in response to type I IFNs and PRR stimulation(7, 29), two features that suggest a role in early antiviral host defense. In support of the importance of IRF1 in host defense are recent reports that rhinovirus and respiratory syncytial virus indirectly suppress IFNλ expression by suppressing IRF1 (30, 31). Using an IRF1-deficient BEAS-2B cell line, we demonstrate that: (1) In response to PRR stimulation, IRF1 supports early expression of IFNβ and IFNλ1/2, STAT1/2 activation, and ISG expression; (2) IRF1 mediates IFN-independent antiviral immunity by supporting constitutive expression of antiviral effectors as well as PRRs that support IRF3 activation; (3) The mechanism by which IRF1 supports gene expression is by modulation of H3K4me1 modification at promoter enhancer regions of IRF1 dependent genes; and (4) That IRF1 modulates expression of TLR2, an unexpected finding that suggests a role for IRF1 in defense against the bacterial pathogens that often co-infect or follow viral respiratory infections.

IRF1 was discovered as a transcription factor that binds to the *IFNB1* promoter region (32, 33). While optimal IFN expression after PRR stimulation may require IRF1, its role in viral infection has been questioned, in part because antiviral defense and IFN expression were not impaired in IRF1 KO mice (11, 34). In support of a role for IRF1 in IFN expression, we found that over-expression of IRF1 in a subset of cells rendered the IRF1-nontransduced cells also resistant to viral infection. Here we also show that while constitutive IRF1 enhances early IFN expression, parent and IRF1 KO cells similarly expressed IFN 24 h after PRR stimulation. It is likely, therefore, that whether the IFN-dependent or -independent functions of IRF1 are relevant to viral defense depends upon cell type, the viral pathogen, infectious dose, and duration of infection. For example, VSV replicates quickly, blocks IFN signaling, and shuts down host translational machinery, so it is unsurprising that even though the parent cells express *IFNB1, IFNL1*, and *IFNL2*, the proteins were undetectable, and that blockade of JAK signaling did not enhance VSV infection. Additionally, our data may apply only to local defenses at target cells for viral infection.

The most prominent mechanism of IFN-independent expression of anti-viral genes is binding of phosphorylated IRF3 to IFN-stimulated response elements (ISRE) in ISG promoter regions, usually, but not always after activation of PRRs such as RIG-I and TLR3 (35–38). IRF family members share sequence homology, including their DNA binding domains (39). Like IRF3 and IRF9, IRF1 binds to DNA elements such as IRE sequences and induces IFN-independent transcription of some ISGs (40, 41). In support of IFN-independent regulation of anti-viral genes, are reports in which ectopic over-expression of IRF1 protects Huh7 cells and STAT1 deficient fibroblasts against a broad range of viruses (8, 40). We show here a second mechanism of IFN-independent ISG expression: regulation of constitutive early expression of antiviral genes.

Constitutive expression of antiviral ISGs is a critical determinant of susceptibility to viral infection, first because it establishes the basal AVS, and second because it reflects the ability to rapidly accelerate ISG transcription through epigenetic mechanisms. *In vivo*, tonic ISG expression may be driven by stimulation of low tonic expression of IFNβ in response to local microbiota (5), or by non-canonical transcription factors (42). This mechanism is not relevant to IRF1 because constitutive expression of types I and III IFN transcripts is identical between parent and IRF1 KO cells, pSTAT1 does not translocate to the nucleus of unstimulated parent or IRF1 KO cells, IRF1 deficiency does not impact expression of STAT1, STAT2, or IRF9 transcripts or proteins (data not shown), and JAK1 inhibition does not increase VSV infection in either parent or IRF1 KO cells (Figure 4F and Supplementary Figure 4).

Rather than these indirect mechanisms, our data demonstrate that IRF1 directly drives constitutive expression of at least three anti-viral genes, *BST2, OAS2*, and *RNASEL*, to maintain an optimal anti-viral state. BST2 (tetherin) is an IFN inducible protein that prevents release of wide range of enveloped viral particles including HIV, VSV, and Ebola (43) by retaining fully formed viral particles inside the cell preventing their spread. Because of its direct interactions, BST2 is also under positive selection and differs among species (44). The OAS family of consists of OAS1, OAS2, and OAS3, which patrol for cytoplasmic dsRNA (45). Upon binding dsRNA, each of the three OAS proteins activate the endoribonuclease RNase L, which indiscriminately degrades cellular and viral RNA (19, 45). While all three OAS family members are ISGs, only OAS2 is transcriptionally regulated by IRF1. OAS family genes are divergent in sequence and all the OAS paralogs as well as RNase L proteins are under constant selective pressure (46, 47). Selective pressure may be higher for OAS2 suggesting higher importance among the three OAS proteins (46). RNase L is regulated by at least two mechanisms: transcriptional regulation by IRF1 and functional responsiveness to OAS2 levels (48). Because of its direct involvement in degrading viral RNAs, domains of RNase L that detect environmental cues and degrade viral RNA have rapidly evolved in mammals (47). Furthermore, by associating with cytoskeletal proteins, RNase L provides a barrier to viral entry independent of its catalytic function (49), thus suggesting an additional mechanism by which IRF1 mediates viral restriction.

IRF1 regulates constitutive expression of antiviral genes is by binding to IREs, distort the conformation of the DNA double helix to allow other transcription factors to access promoter regions. In support, Karwacz and colleagues recently demonstrate that IRF1 deficiency decreases activating histone marks such as H3K9Ac and H3K4me3 to limit access of transcription factors such as STAT1 and KLF7 (29). Additionally, IRF1 modulates chromatin landscape with BATF1 to regulate type 1 regulatory T cell differentiation. The transcription factor BRD4 and the transcription elongation factor P-TEFb are recruited together to the promoter region of ISGs to stimulate their expression while P-TEFb and BRD4 are not constitutively present at the ISG promoters before IFN stimulation (50). Here we show that IRF1 modulates H3K4 monomethylation at the promotor regions of target genes to maintain constitutive expression and upon IFN stimulation, cooperates with BRD4 for optimal level of expression–two critical parameters of the AVS.

In addition to mediating IRF3 signaling and constitutive ISG expression, IRF1 also mediates constitutive expression of TLR3, and TLR3 signaling is deficient in IRF1 KO BEAS-2B respiratory epithelial cells. TLR3 is an important PRR that recognizes viral PAMPs early during infection because it is localized to early endosomes, which are hijacked by viruses for cellular entry. Since TLR3 activates IRF3, modulation of TLR3 expression applies to both IFN-dependent and independent antiviral defense. However, studies showing transcriptional or positional regulation of TLR3 are limited. Recently, Sun and colleagues reported that *TLR3* is regulated by a complex of IRF1, IRF2 and host cell factor C2 (HCFC2) in murine monocytic cells (51). However, patterns of TLR3 expression are cell and species dependent (52, 53). For example, unlike murine monocytic cells, types I and III IFNs do not enhance expression of TLR3 in epithelial cells. By contrast, Bose and coworkers showed that S100A9 regulates intracellular trafficking of TLR3 in murine bone marrow derived macrophages (20). Since expression of *S100A9* is decreased and TLR3 poorly localizes to the endosomes in IRF1 KO cells, maintaining proper TLR3 localization is another method by which IRF1 enhances anti-viral defenses.

In conclusion, our study highlights a critical role for IRF1 in regulating constitutive antiviral gene networks to confer resistance against viral infections in human respiratory epithelial cells. IRF1 prominently participates in antiviral defense by regulating early expression of IFNs and maintaining histone H3K4me1 marks at gene promoter/enhancer regions in homeostatic conditions. In addition to antiviral defense, IRF1 participates in antibacterial defense, autoimmunity, tumor immune surveillance, proinflammatory disease and immune system development, suggesting broad implications for the functional and mechanistic data described in this report.

## Materials and Methods

### Cell Line, Virus and Reagents

Human bronchial epithelial cells (BEAS-2B, ATCC CRL-9609) were cultured in BEGM Bronchial Epithelial Cell Growth Medium (Lonza, Walkersville, MD) supplemented with BEGM Bullet Kit. A549 cells (ATCC CCL 185) were cultured in F12K medium supplemented with 10% fetal bovine serum. IRF1 KO cells were custom developed using a CRISPR/Cas gene editing method by Applied StemCell (Milpitas, CA). IRF1 KO A549 was developed in-house using CRISPR/Cas9 gene editing method using guideRNAs from GenScript. Recombinant VSV-GFP was propagated in Vero cells when the cells were >80% confluent. Influenza viruses were grown in MDCK cells. Virus titer for the stock was determined by plaque assay. Human IFNβ1b was purchased from PBL assay science (catalog # 11420-1). Human IFNλ1 (1598-IL) was purchased from R&D Systems (Minneapolis, MN). Ruxolitinib (catalog # S1378), AZD1480 (catalog # S2162), and PFI-1 (catalog # S1216) were purchased from Selleck Chemicals (Houston, TX). The transfection reagents Lyovec (catalog # lyec), Fugene6 (catalog # E2691), Lipofectamine RNAiMAX (catalog #56532), and XtremeGene 9, (catalog # 23644700) were purchased from Invivogen (San Diego, CA), Promega (Madison, WI), Thermo Fisher Scientific (Waltham, MA), and Roche (San Francisco, CA), respectively. LPS (L2630) and PAM2CSK4 (tlrl-pm2s-1) were purchased from Sigma (Saint Louis, MO) and Invivogen, respectively. The following commercially available antibodies for western blot were purchased from Cell Signaling Technologies (Danvers, MA). IRF1 (8478), P-IRF3 S396 (4947), IRF3 (4302), P-STAT1 Y701 (7649), STAT1 (9172), P-TBK1 S172 (5483), TBK1 (3504), EEA1 (3288). P-NFKBIA (ab92700) antibody was purchased from Abcam (Cambridge, MA). TLR3 (14-9039-82) and TLR2 (14-9039-82) antibodies were purchased from Thermo Scientific. Anti-BRD4 (A700-004) antibody was purchased from Bethyl Research Laboratory. Antibodies against GFP (sc-9996), Actin (sc-47778), anti-rabbit IgG (sc-2054), and anti-mouse IgG (sc-2055) were purchased from Santa Cruz Biotechnology (Dallas, TX). Silencer Select siRNAs were purchased from Thermo Fisher Scientific.

### Plaque Assays

Virus dilutions of rVSV were added to confluent Vero monolayers in 12-well plates. The plates were incubated for 1 h at 37°C and rocked at 15-min intervals. Virus inoculum was then removed, and the cells were overlaid with 0.5% methylcellulose to allow the plaques to develop. Plaques were fixed with 4% formaldehyde and stained with crystal violet and counted.

### Generation of Lentivirus Pseudoparticles Expressing IRF1

Bicistronic lentiviruses expressing tagRFP and IRF1were generated as described before (8). A virus expressing fire fly luciferase and tagRFP was generated to be used as a control. Viruses were titrated in HEK293T cells and percent cells infected was calculated by fluorescence microscopy.

### SiRNA Transfection of OAS2, BST2, and RNASEL

Parent BEAS-2B cells were reverse transfected with siRNAs for OAS2, BST2, and RNASEL using Lipofectamine RNAiMAX (Invitrogen). The cells were plated at 1.25 × 10^5^ cells/ml. The nucleic acid and transfection reagent complex were assembled using 4 μl of OAS2, BST2, or RNASEL siRNA (final concentration of 20 nM) and 4 μl of Lipofectamine RNAiMAX. A mixture of two negative controls siRNAs (silencer select negative control #1 and #2 from Thermo Fisher Scientific) were used. The mix was added dropwise to each well and the media was changed after 24 h.

### Western Blot Analysis

Cells were scarped and harvested in ice-cold PBS, clarified by centrifugation, and cell pellet was lysed using RIPA buffer (89901) containing protease and phosphatase inhibitors (88666, 88667) from Thermo Fisher Scientific. Protein concentration was calculated using a Bradford assay following the manufacturer's protocol. Lysates were stored at −80°C until further use. Cell lysates were electrophoresed using a 10 or 12% Novex Bis-Tris precast gel (Thermo Fisher Scientific). Protein was then immobilized on PVDF or nitrocellulose membrane (Thermo Fisher Scientific) using an iBlot 2 gel transfer device.

Membranes were blocked with 5% non-fat milk in 1X Tris-HCl buffer saline with 0.5% Tween-20 (TBST) for 1 h. The membrane was processed for overnight incubation at 4 °C with primary antibodies diluted in blocking buffer. For the pIRF3 immunoblot, the membrane was incubated with diluted primary antibody in 5% w/v BSA in TBS containing 0.05% Tween-20 at 4°C. Membranes were washed three times in Tris-buffered saline to remove excess antibody. Membrane was then incubated in appropriate secondary antibody conjugated to horseradish peroxidase.

### Fluorescence-Activated Cell Sorting (FACS)

BEAS-2B cells were trypsinized, fixed in 4% paraformaldehyde, washed with PBS and re-suspended in 1X PBS with 1% fetal bovine serum (FBS). Single cell suspensions were processed for flow cytometry using BD Biosciences LSRII and the data were analyzed using Flowjo software (Ashland, OR).

### Immunofluorescence Microscopy

BEAS-2B cells were plated on glass coverslips. Cells were fixed with 4% PFA for 15 min at RT, permeabilized with 0.1% Triton-X 100 in 1X PBS for 10 min and stained with appropriate primary and secondary antibodies. Coverslips were mounted on glass slides and imaged using Zeiss Axio Observer Z1 with a photometric evolve 512 camera using a 63X objective lens.

### Quantitative Fluorescent Microscopy

Cells were fixed with 4% PFA for 15 min at room temperature and washed with PBS twice. The cells were permeabilized with 0.1% triton-X 100 (Sigma) in 1X PBS for 10 min, nuclei were then stained with Hoechst 33342 (Life Technologies) and imaged with the Celigo Imaging Cytometer (Nexcelom, Lawrence, MA).

### RT-qPCR

Total RNA was extracted using a RNeasy kit (Qiagen) following manufacturer's protocol. Verso cDNA Synthesis Kit (Thermo Scientific) was used for reverse transcription following manufacturer's protocol. PCR was performed in triplicate using the Applied Biosystems Power SYBR Green Power Mastermix (ThermoFisher Scientific) using Quantstudio 12K flex instrument (ThermoFisher Scientific). Primers used in the assays are listed in Table 1. Data were analyzed by the 2^−ΔΔ*Cq*^ method, where *C*_*q*_ is threshold cycle, and normalized to UBC or GAPDH mRNA. Data are represented as levels of mRNA relative to that of the control samples and are displayed as the means ± SEM of results from at least three independent experiments. Expression of IFN transcripts were examined as reported previously (54).

**Table 1:** List of primers used in this study.

**Primer**	**Forward sequence**	**Reverse sequence**
*RNASEL*	AAGCCGCTGTGTATGGTAAG	CGCTCTTGATCCTCCTTTGT
*TLR3*	GGATAGCTCTCCTTCACCATTC	GACCCTCCAACATGTCATCAT
*TLR2*	TGATGCTGCCATTCTCATTCT	CAGGTAGGTCTTGGTGTTCATT
*TLR4*	CCTATCAGGGCTGTGTGTATTT	TCTCAAACAGCCATAGACATCC
*OAS1*	TGCGCTCAGCTTCGTACTGA	GGTGGAGAACTCGCCCTCTT
*OAS2*	GAGTGGCCATAGGTGGCTC	CGAGGATGTCACGTTGGCTT
*OAS3*	GATGAGGGAGTGGGTCTATCT	TGGAGAGTCAGGCTGTCTAA
*MX1*	CAATCAGCCTGCTGACATTG	TGTCTCCTGCCTCTGGATG
*BST2*	GGAAGCTGGCACATCTTGGA	CTAACCGTGTTGCCCCATGA
*IRF1*	CCAAGAGGAAGTCATGTG	TAGCCTGGAACTGTGTAG
*IRF8*	AATGCAAACTAGGCGTGGCA	TAATCGTCCACAGAAGGCTCC
*IRF9*	GTGCTGGGATGATACAGCTAAG	CAGGCGAGTCTTCCAGACAG
*ISG15*	TCCTGGTGAGGAATAACAAGGG	GTCAGCCAGAACAGGTCGTC
*IFIT1*	GAATGAAGCCCTGGAGTACTATG	GCTGATATCTGGGTGCCTAAG
VSV N	GCAGGTTTGTTGTACGCTTATG	TCGTCAATCCTCCGGTACTAT
Perth H3N2 HA	AAAGCACTCAAGCAGCAATCG	TCTCCAGGGCAACAAGAAGC
New Caledonia 99 H1N1 HA	GCTTATGTCTCTGTAGTGTCT	TAGTTGATTCTTCCTTCCTGAT
Brisbane 07 H1N1 HA	TGTGATGCGAAGTGCCAAAC	AATGGATGGGATGTTCCTTAGTCC
California 09 H1N1 HA	AGAAGGGAGAATGAACTATTACTGGAC	CGTGGACTGGTGTATCTGAAATG
PR8 Matrix	CTTCTAACCGAGGTCGAAACGTA	GGTGACAGGATTGGTCTTGTCTTTA
IRF9 ChIP	ACATGGGTCTCTGGGTATCT	TTCATCCCTGCTGAGTGTTC
IFIT1 ChIP	GACAAATGCAAACTGGCTGAA	ACACAGCTACTGCTCTTTGG
MX1 ChIP	CACTGCCCCCTCGTCGTGGCACCGC	TTTCTGCTCGCTGGTTTCCAGA
BST2 ChIP	CTTGGGCCCTTCCCAGCTGGGT	GCCTCTGCCTCTTCAGGTCATA
*GAPDH*	GAGTCAACGGATTTGGTC	GGTGGAATCATATTGGAACAT
*UBC*	ATTTGGGTCGCAGTTCTTG	TGCCTTGACATTCTCGATGGT
*SDHA*	TGGGAACAAGAGGGCATCTG	CCACCACTGCATCAAATTCATG

### Luciferase Assay

Parent and IRF1 KO cells were transfected with a plasmid expressing Firefly luciferase under the control of IFNβ promoter and a plasmid constitutively expressing Renilla luciferase. Cells were then transfected with poly I:C and processed using Dual Glo luciferase assay kit (Promega) as per the manufacturer's instruction. Relative light unit was examined using a Glowmax Navigator Microplate Luminometer (Promega) at 6 h and 24 h. Firefly luciferase expression was normalized to Renilla luciferase expression.

### Transcriptome Analysis

BEAS-2B cells were either left untreated or treated with 0.2 ng/ml of IFNβ and 5 ng/ml of IFNλ1 for 24 h and RNA was isolated using RNeasy spin columns including on-column DNase digestion (Qiagen,Germantown, MD). RNA integrity was analyzed using a bioanalyzer (Agilent, Santa Clara, CA) and samples with RIN > 8 were further considered for RNA sequencing. To obtain the final sequencing library, 14 cycles of PCR were performed using Phusion Hot Start High-Fidelity DNA Polymerase (Finnzymes, Espoo, Finland). Three biological replicates were used and for each sample replicate we obtained ~50 million paired 50-mer reads (using the Illumina HiSeq 2,500 platform, Illumina, San Diego, CA). Data were examined using Tuxedo pipeline for differential gene expression analysis. We used default parameters for these software programs and used a cutoff of 0.05 False Discovery Rate (FDR) by Benjamin-Hochberg method to determine differentially expressed genes with statistical significance. Transcriptome data have been deposited in the NCBI Gene Expression Omnibus under accession number GSE114284.

### Bioinformatics Analysis

Scatter plots and volcano plots of RNA-Seq read counts for all representative genes from the Cufflinks analyses in parent and IRF1 knockout cells were developed using Tuxedo pipeline and R. Network analysis was done using Ingenuity Pathway Analysis (Qiagenbioinformatics.com). Heatmaps were generated using Morpheus web platform from Broad Institute. Venn diagram was generated using BioVenn web platform.

### Chromatin Immunoprecipitation

The following antibodies were used in qChIP experiments: anti-IRF1 (8478S; Cell Signaling Technology), anti-H3K4me1 (ab8895; Abcam) and anti-BRD4 (In house). Briefly, cells on a 15-cm plate were treated with 1% formaldehyde in Covaris fixing buffer for 5 min at room temperature followed by 0.125 M Glycine for 5 min. After washing three times with 1x ice cold PBS (pH 7.4), cells were lysed in 1 ml of the ChIP lysis buffer to isolate nuclei and nuclei were then sonicated in Covaris® M220 Focused-ultrasonicator™ to shear the genomic DNA into 200–700-bp fragments. After centrifugation, 200 μl of supernatants were diluted with 900 μl of the ChIP dilution buffer. After pre-clearing, magnetic protein A-beads (Dynabeads protein G, Thermo Fisher Scientific) were incubated with the antibodies-chromatin mix for 3 h. Precleared chromatin was incubated with an appropriate antibody overnight at 4°C with rotation. Precipitates were then washed twice with each of the following buffers in order: low salt wash buffer, high salt wash buffer, LiCl wash buffer, and TE buffer. After elution of the chromatin complexes, the cross-link was reversed, and RNA and proteins were digested with RNase and proteinase K, respectively. DNA was then recovered by phenol/chloroform extraction followed by ethanol precipitation and resuspended in 50 μl of 10 mm Tris, pH 8. Five μl of each sample were used for quantification of the specific region of genomic DNA (100–150 bp) by duplicate real-time PCR amplifications. Input DNA (1%) was used for normalization.

### Standard Biosecurity and Institutional Safety Procedures

We followed appropriate standard biosecurity and institutional safety procedures.

### Statistical Analyses

Statistical analyses were done using GraphPad Prism (La Jolla, CA). We used *t*-tests and ANOVA where appropriate.

## Author Contributions

DP and RR conceived and designed the study. DP, EG, MB, and HN performed the experiments. DP, MB, KO, and RR analyzed the results. DP and RR wrote the manuscript. All authors contributed to manuscript revision, read and approved the submitted version. ES contributed to generation of results added in the revised manuscript. She also participated in writing the revised manuscript.

### Conflict of Interest Statement

The authors declare that the research was conducted in the absence of any commercial or financial relationships that could be construed as a potential conflict of interest.

## References

[1] Schoggins JW. Interferon-stimulated genes: roles in viral pathogenesis. Curr Opin Virol. (2014) 6:40–6. 10.1016/j.coviro.2014.03.006 24713352 PMC4077717

[2] Schneider WM Chevillotte MD Rice CM. Interferon-stimulated genes: a complex web of host defenses. Annu Rev Immunol. (2014) 32:513–45. 10.1146/annurev-immunol-032713-120231 24555472 PMC4313732

[3] Xu L Wang W Peppelenbosch MP Pan Q Non-canonical antiviral mechanisms of ISGs: dispensability of inducible interferons. Trends Immunol. (2017) 38:1–2. 10.1016/j.it.2016.11.002 27916385

[4] Blaszczyk K Nowicka H Kostyrko K Antonczyk A Wesoly J Bluyssen HA. The unique role of STAT2 in constitutive and IFN-induced transcription and antiviral responses. Cytokine Growth Factor Rev. (2016) 29:71–81. 10.1016/j.cytogfr.2016.02.010 27053489

[5] Gough DJ Messina NL Clarke CJ Johnstone RW Levy DE. Constitutive type I interferon modulates homeostatic balance through tonic signaling. Immunity. (2012) 36:166–74. 10.1016/j.immuni.2012.01.011 22365663 PMC3294371

[6] Paludan SR. Innate antiviral defenses Independent of Inducible IFNalpha/beta production. Trends Immunol. (2016) 37:588–96. 10.1016/j.it.2016.06.003 27345728

[7] Novatt H Theisen TC Massie T Massie T Simonyan V Voskanian-Kordi A. Distinct patterns of expression of transcription factors in response to interferonbeta and interferonlambda1. J Interferon Cytokine Res. (2016) 36:589–98. 10.1089/jir.2016.0031 27447339

[8] Schoggins JW Wilson SJ Panis M Murphy MY Jones CT Bieniasz P. A diverse range of gene products are effectors of the type I interferon antiviral response. Nature. (2011) 472:481–5. 10.1038/nature09907 21478870 PMC3409588

[9] Mboko WP Mounce BC Emmer J Darrah E Patel SB Tarakanova VL. Interferon regulatory factor 1 restricts gammaherpesvirus replication in primary immune cells. J Virol. (2014) 88:6993–7004. 10.1128/JVI.00638-14 24719409 PMC4054362

[10] Nair S Poddar S Shimak RM Diamond MS Interferon regulatory factor-1 (IRF-1) protects against chikungunya virus induced immunopathology by restricting infection in muscle cells. J Virol. (2017) 91 e01419–17 10.1128/JVI.01419-17 PMC5660487 28835505

[11] Reis LF Ruffner H Stark G Aguet M Weissmann C. Mice devoid of interferon regulatory factor 1 (IRF-1) show normal expression of type I interferon genes. EMBO J. (1994) 13:4798–806. 10.1002/j.1460-2075.1994.tb06805.x 7957048 PMC395418

[12] Kimura T Nakayama K Penninger J Kitagawa M Harada H Matsuyama T. Involvement of the IRF-1 transcription factor in antiviral responses to interferons. Science. (1994) 264:1921–4. 10.1126/science.8009222 8009222

[13] Maloney NS Thackray LB Goel G Hwang S Duan E Vachharajani P. Essential cell-autonomous role for interferon (IFN) regulatory factor 1 in IFN-gamma-mediated inhibition of norovirus replication in macrophages. J Virol. (2012) 86:12655–64. 10.1128/JVI.01564-12 22973039 PMC3497668

[14] Voigt E Inankur B Baltes A Yin J. A quantitative infection assay for human type I, II, and III interferon antiviral activities. Virol J. (2013) 10:224. 10.1186/1743-422X-10-224 23829314 PMC3716869

[15] Vogel SN Friedman RM Hogan MM. Measurement of antiviral activity induced by interferons alpha, beta, and gamma. Curr Protoc Immunol. (2001) 6:9. 10.1002/0471142735.im0609s37 18432822

[16] Weidner JM Jiang D Pan XB Chang J Block TM Guo JT. Interferon-induced cell membrane proteins, IFITM3 and tetherin, inhibit vesicular stomatitis virus infection via distinct mechanisms. J Virol. (2010) 84:12646–57. 10.1128/JVI.01328-10 20943977 PMC3004348

[17] Li Y Banerjee S Wang Y Goldstein SA Dong B Gaughan C. Activation of RNase L is dependent on OAS3 expression during infection with diverse human viruses. Proc Natl Acad Sci USA. (2016) 113:2241–6. 10.1073/pnas.1519657113 26858407 PMC4776461

[18] Drappier M Michiels T. Inhibition of the OAS/RNase L pathway by viruses. Curr Opin Virol. (2015) 15:19–26. 10.1016/j.coviro.2015.07.002 26231767 PMC7185432

[19] Chakrabarti A Jha BK Silverman RH. New insights into the role of RNase L in innate immunity. J Interferon Cytokine Res. (2011) 31:49–57. 10.1089/jir.2010.0120 21190483 PMC3021357

[20] Tsai SY Segovia JA Chang TH Shil NK Pokharel SM Kannan TR. Regulation of TLR3 activation by S100A9. J Immunol. (2015) 195:4426–37. 10.4049/jimmunol.1500378 26385519 PMC4747058

[21] Menager P Roux P Megret F Bourgeois JP Le Sourd AM Danckaert A. Toll-like receptor 3 (TLR3) plays a major role in the formation of rabies virus Negri Bodies. PLoS Pathog. (2009) 5:e1000315. 10.1371/journal.ppat.1000315 19247444 PMC2642728

[22] Heinz S Romanoski CE Benner C Glass CK. The selection and function of cell type-specific enhancers. Nat Rev Mol Cell Biol. (2015) 16:144–54. 10.1038/nrm3949 25650801 PMC4517609

[23] Calo E Wysocka J. Modification of enhancer chromatin: what, how, and why? Mol Cell. (2013) 49:825–37. 10.1016/j.molcel.2013.01.038 23473601 PMC3857148

[24] Leung YT Shi L Maurer K Song L Zhang Z Petri M. Interferon regulatory factor 1 and histone H4 acetylation in systemic lupus erythematosus. Epigenetics. (2015) 10:191–9. 10.1080/15592294.2015.1009764 25611806 PMC4622916

[25] Tian B Yang J Zhao Y Ivanciuc T Sun H Garofalo RP. BRD4 Couples NF-kappaB/RelA with airway inflammation and the IRF-RIG-I amplification loop in respiratory syncytial virus infection. J Virol. (2017) 91:e00007–17. 10.1128/JVI.00007-17 28077651 PMC5331805

[26] Subauste MC Jacoby DB Richards SM Proud D. Infection of a human respiratory epithelial cell line with rhinovirus. Induction of cytokine release and modulation of susceptibility to infection by cytokine exposure. J Clin Invest. (1995) 96:549–57. 10.1172/JCI118067 7615827 PMC185229

[27] Reddel RR Ke Y Gerwin BI McMenamin MG Lechner JF Su RT Transformation of human bronchial epithelial cells by infection with SV40 or adenovirus-12 SV40 hybrid virus, or transfection via strontium phosphate coprecipitation with a plasmid containing SV40 early region genes. Cancer Res. (1988) 48:1904–9. 2450641

[28] Spurrell JC Wiehler S Zaheer RS Sanders SP Proud D. Human airway epithelial cells produce IP-10 (CXCL10) *in vitro* and *in vivo* upon rhinovirus infection. Am J Physiol Lung Cell Mol Physiol. (2005) 289:L85–95. 10.1152/ajplung.00397.2004 15764644

[29] Karwacz K Miraldi ER Pokrovskii M Madi A Yosef N Wortman I. Critical role of IRF1 and BATF in forming chromatin landscape during type 1 regulatory cell differentiation. Nat Immunol. (2017) 18:412–21. 10.1038/ni.3683 28166218 PMC5901650

[30] Ueki IF Min-Oo G Kalinowski A Ballon-Landa E Lanier LL Nadel JA. Respiratory virus-induced EGFR activation suppresses IRF1-dependent interferon lambda and antiviral defense in airway epithelium. J Exp Med. (2013) 210:1929–36. 10.1084/jem.20121401 23999497 PMC3782052

[31] Kalinowski A Galen BT Ueki IF Sun Y Mulenos A Osafo-Addo A. Respiratory syncytial virus activates epidermal growth factor receptor to suppress interferon regulatory factor 1-dependent interferon-lambda and antiviral defense in airway epithelium. Mucosal Immunol. (2018) 11:958–67. 10.1038/mi.2017.120 29411775 PMC6431552

[32] Harada H Fujita T Miyamoto M Kimura Y Maruyama M Furia A. Structurally similar but functionally distinct factors, IRF-1 and IRF-2, bind to the same regulatory elements of IFN and IFN-inducible genes. Cell. (1989) 58:729–39. 10.1016/0092-8674(89)90107-4 2475256

[33] Miyamoto M Fujita T Kimura Y Maruyama M Harada H Sudo Y. Regulated expression of a gene encoding a nuclear factor, IRF-1, that specifically binds to IFN-beta gene regulatory elements. Cell. (1988) 54:903–13. 10.1016/S0092-8674(88)91307-4 3409321

[34] Matsuyama T Kimura T Kitagawa M Pfeffer K Kawakami T Watanabe N. Targeted disruption of IRF-1 or IRF-2 results in abnormal type I IFN gene induction and aberrant lymphocyte development. Cell. (1993) 75:83–97. 10.1016/0092-8674(93)90681-F 8402903

[35] Yoneyama M Suhara W Fukuhara Y Fukuda M Nishida E Fujita T. Direct triggering of the type I interferon system by virus infection: activation of a transcription factor complex containing IRF-3 and CBP/p300. EMBO J. (1998) 17:1087–95. 10.1093/emboj/17.4.1087 9463386 PMC1170457

[36] Grandvaux N Servant MJ tenOever B Sen GC Balachandran S Barber GN. Transcriptional profiling of interferon regulatory factor 3 target genes: direct involvement in the regulation of interferon-stimulated genes. J Virol. (2002) 76:5532–9. 10.1128/JVI.76.11.5532-5539.2002 11991981 PMC137057

[37] Paladino P Cummings DT Noyce RS Mossman KL. The IFN-independent response to virus particle entry provides a first line of antiviral defense that is independent of TLRs and retinoic acid-inducible gene I. J Immunol. (2006) 177:8008–16. 10.4049/jimmunol.177.11.8008 17114474

[38] DeWitte-Orr SJ Mehta DR Collins SE Suthar MS Gale M Jr Mossman KL. Long double-stranded RNA induces an antiviral response independent of IFN regulatory factor 3, IFN-beta promoter stimulator 1, and IFN. J Immunol. (2009) 183:6545–53. 10.4049/jimmunol.0900867 19864603 PMC2885285

[39] Taniguchi T Ogasawara K Takaoka A Tanaka N. IRF family of transcription factors as regulators of host defense. Annu Rev Immunol. (2001) 19:623–55. 10.1146/annurev.immunol.19.1.623 11244049

[40] Xu L Zhou X Wang W Wang Y Yin Y Laan LJ. IFN regulatory factor 1 restricts hepatitis E virus replication by activating STAT1 to induce antiviral IFN-stimulated genes. FASEB J. (2016) 30:3352–67. 10.1096/fj.201600356R 27328944

[41] Nair S Michaelsen-Preusse K Finsterbusch K Stegemann-Koniszewski S Bruder D Grashoff M. Interferon regulatory factor-1 protects from fatal neurotropic infection with vesicular stomatitis virus by specific inhibition of viral replication in neurons. PLoS Pathog. (2014) 10:e1003999. 10.1371/journal.ppat.1003999 24675692 PMC3968136

[42] Wang W Yin Y Xu L Su J Huang F Wang Y. Unphosphorylated ISGF3 drives constitutive expression of interferon-stimulated genes to protect against viral infections. Sci Signal. (2017) 10:eaah4248. 10.1126/scisignal.aah4248 28442624

[43] Malim MH Bieniasz PD. HIV restriction factors and mechanisms of evasion. Cold Spring Harb Perspect Med. (2012) 2:a006940. 10.1101/cshperspect.a006940 22553496 PMC3331687

[44] Evans DT Serra-Moreno R Singh RK Guatelli JC. BST-2/tetherin: a new component of the innate immune response to enveloped viruses. Trends Microbiol. (2010) 18:388–96. 10.1016/j.tim.2010.06.010 20688520 PMC2956607

[45] Hornung V Hartmann R Ablasser A Hopfner KP. OAS proteins and cGAS: unifying concepts in sensing and responding to cytosolic nucleic acids. Nat Rev Immunol. (2014) 14:521–8. 10.1038/nri3719 25033909 PMC7097587

[46] Mozzi A Pontremoli C Forni D Clerici M Pozzoli U Bresolin N. OASes and STING, adaptive evolution in concert. Genome Biol Evol. (2015) 7:1016–32. 10.1093/gbe/evv046 25752600 PMC4419793

[47] Jin W Wu DD Zhang X Irwin DM Zhang YP. Positive selection on the gene RNASEL: correlation between patterns of evolution and function. Mol Biol Evol. (2012) 29:3161–8. 10.1093/molbev/mss123 22513284

[48] Birdwell LD Zalinger ZB Li Y Wright PW Elliott R Rose KM. Activation of RNase L by murine coronavirus in myeloid cells is dependent on basal oas gene expression and independent of virus-induced interferon. J Virol. (2016) 90:3160–72. 10.1128/JVI.03036-15 26739051 PMC4810646

[49] Ezelle HJ Malathi K Hassel BA. The Roles of RNase-L in antimicrobial immunity and the cytoskeleton-associated innate response. Int J Mol Sci. (2016) 17:E74. 10.3390/ijms17010074 26760998 PMC4730318

[50] Patel MC Debrosse M Smith M Dey A Huynh W Sarai N. BRD4 coordinates recruitment of pause release factor P-TEFb and the pausing complex NELF/DSIF to regulate transcription elongation of interferon-stimulated genes. Mol Cell Biol. (2013) 33:2497–507. 10.1128/MCB.01180-12 23589332 PMC3700095

[51] Sun L Jiang Z Acosta-Rodriguez VA Berger M Du X Choi JH. HCFC2 is needed for IRF1- and IRF2-dependent Tlr3 transcription and for survival during viral infections. J Exp Med. (2017) 214:3263–77. 10.1084/jem.20161630 28970238 PMC5679162

[52] Heinz S Haehnel V Karaghiosoff M Schwarzfischer L Muller M Krause SW. Species-specific regulation of Toll-like receptor 3 genes in men and mice. J Biol Chem. (2003) 278:21502–9. 10.1074/jbc.M301476200 12672806

[53] Rehli M. Of mice and men: species variations of Toll-like receptor expression. Trends Immunol. (2002) 23:375–8. 10.1016/S1471-4906(02)02259-7 12133792

[54] Hillyer P Mane VP Schramm LM Puig M Verthelyi D Chen A. Expression profiles of human interferon-alpha and interferon-lambda subtypes are ligand- and cell-dependent. Immunol Cell Biol. (2012) 90:774–83. 10.1038/icb.2011.109 22249201 PMC3442264

