# Supplementary material for: IRF1 Maintains Optimal Constitutive Expression of Antiviral Genes and Regulates the Early Antiviral Response
Source: Front Immunol. 2019 May 15;10:1019. doi: 10.3389/fimmu.2019.01019 (PMC6529937; doi:10.3389/fimmu.2019.01019)
Supplement: Supplementary file 2 [file Data_Sheet_1_.pdf]

Supplementary Figure 1

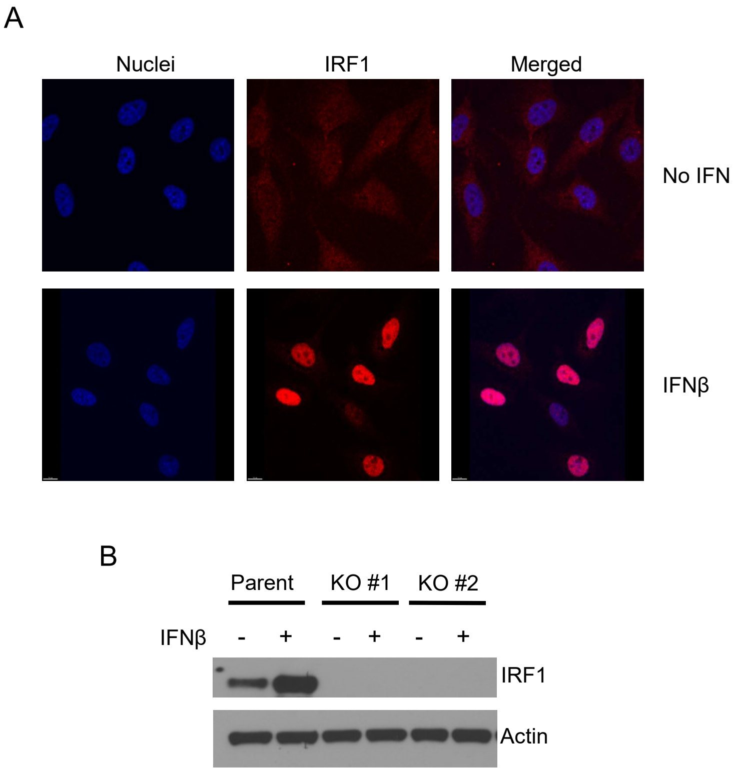

Supplementary Figure 2

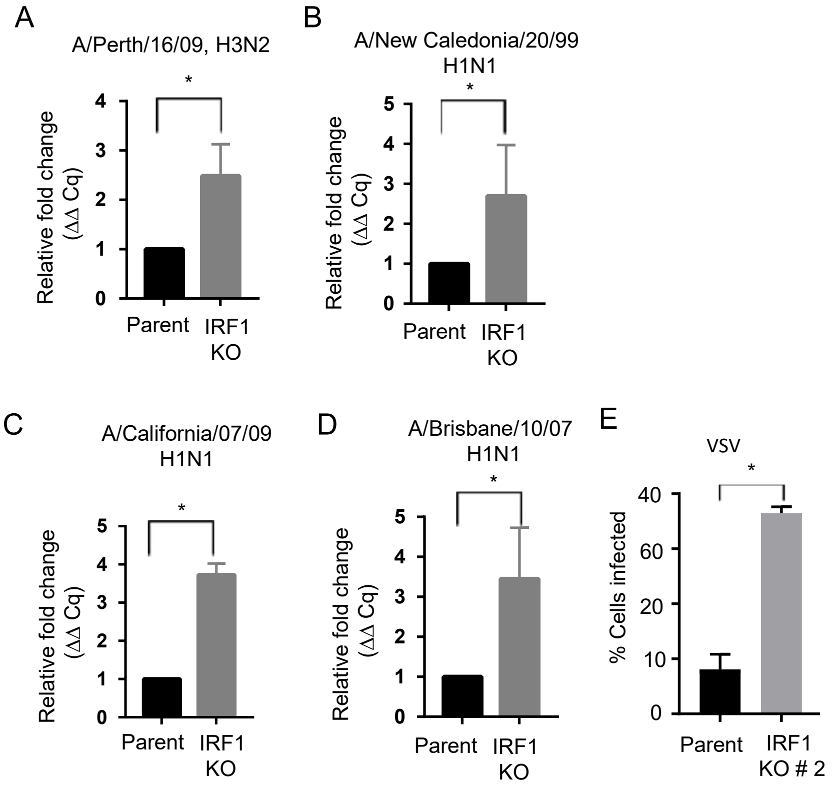

Supplementary Figure 3

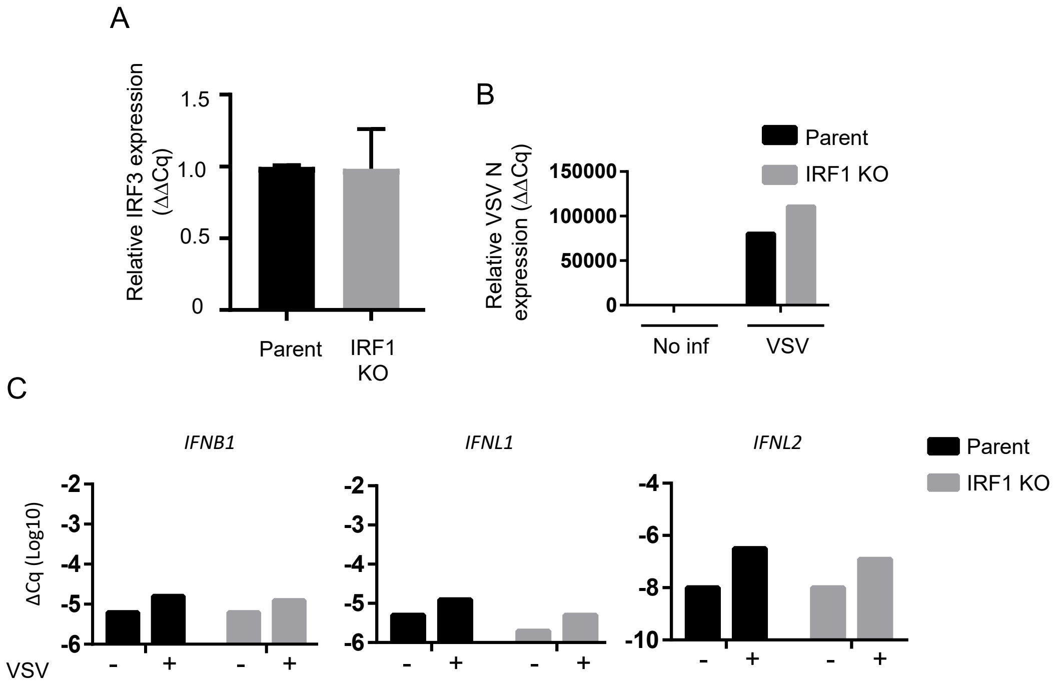

Supplementary Figure 4

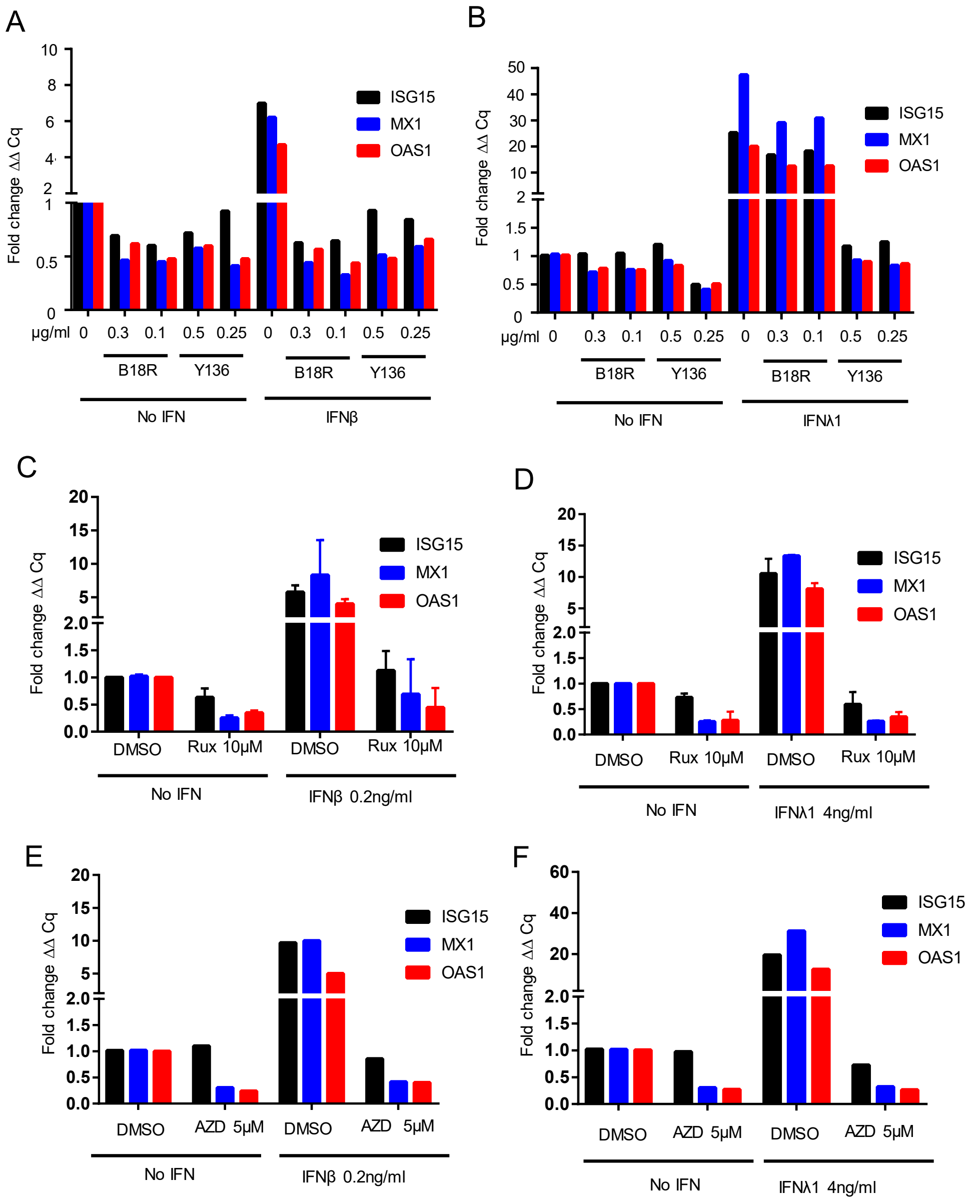

Supplementary Figure 5

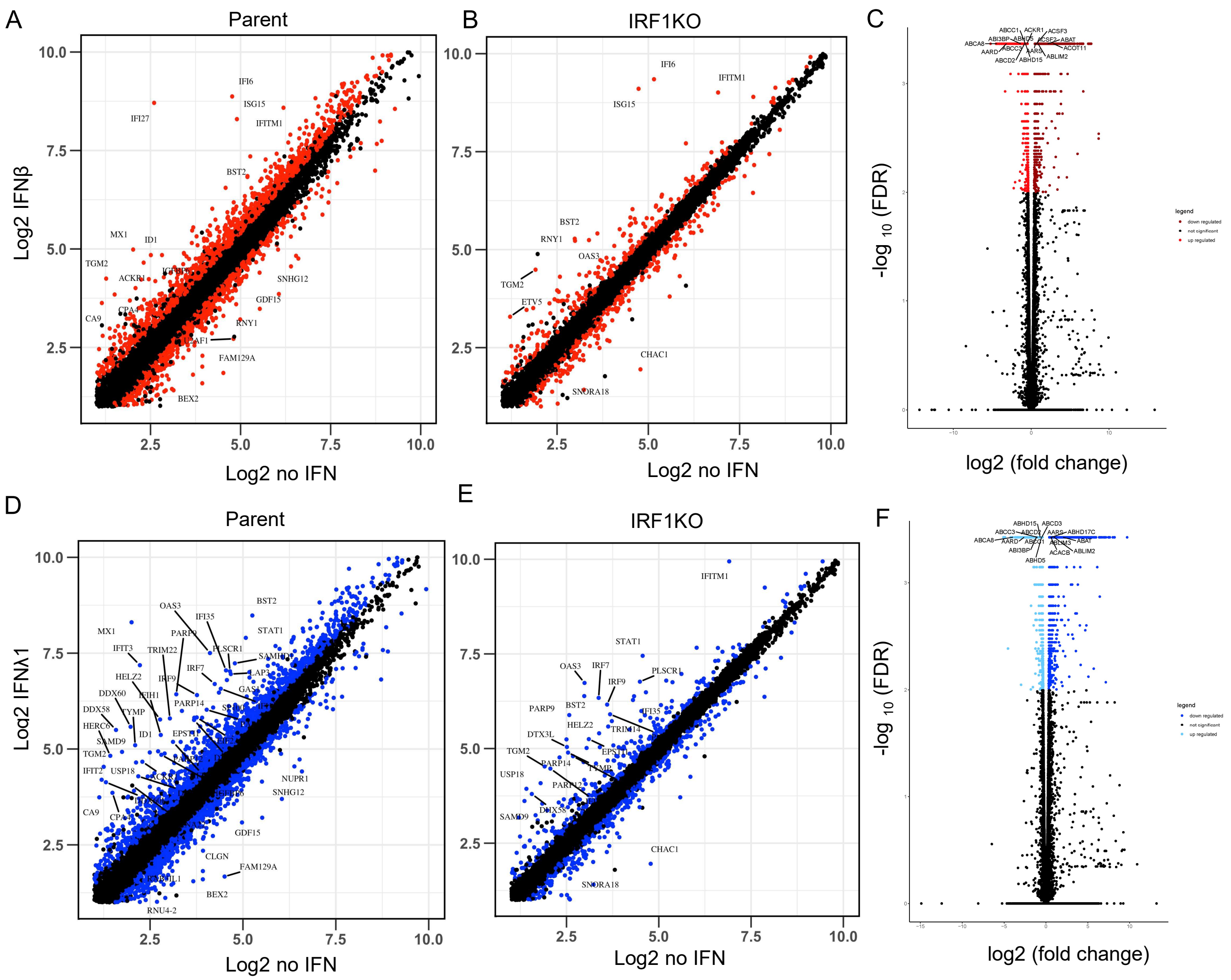

Supplementary Figure 6

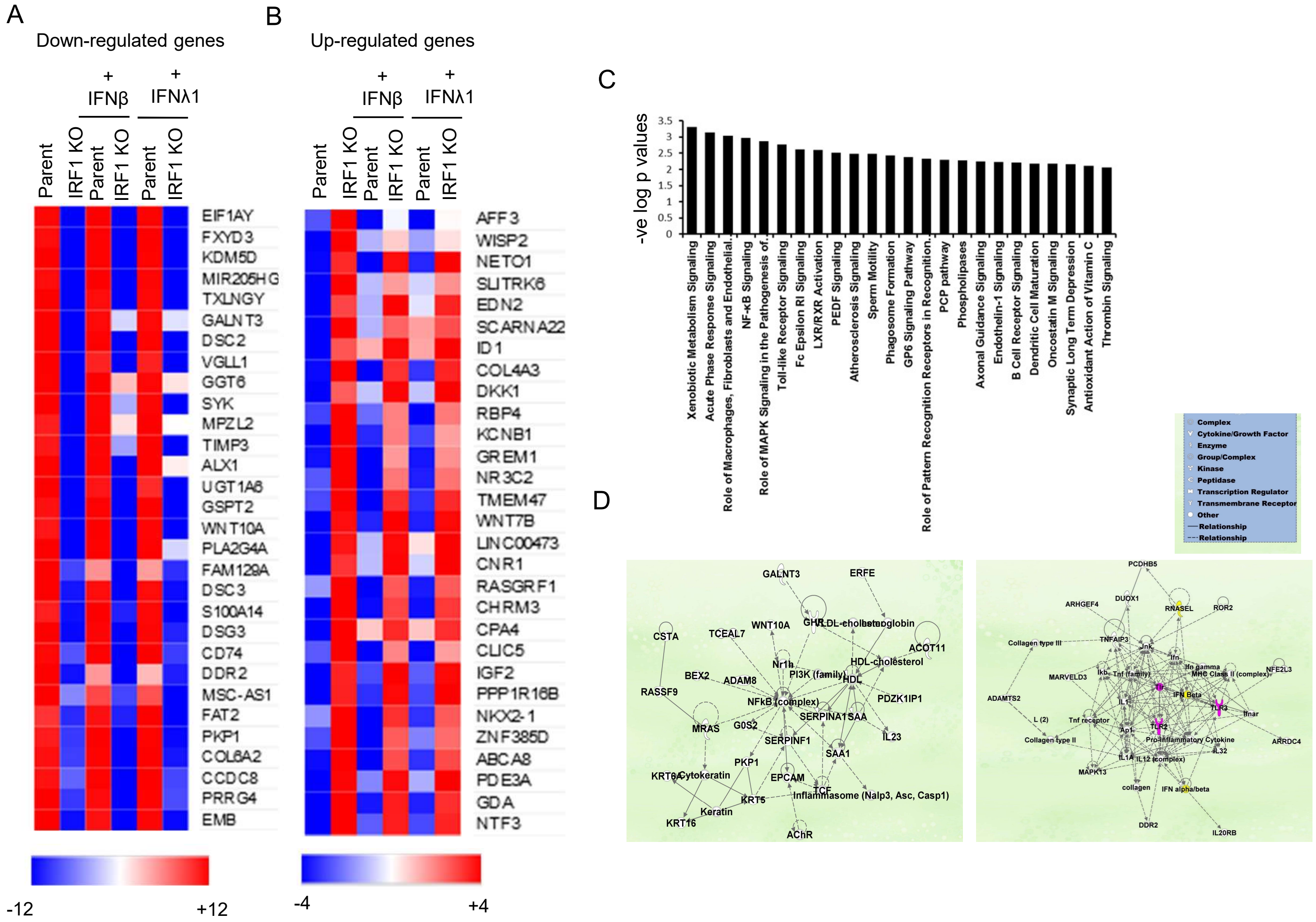

Supplementary Figure 7

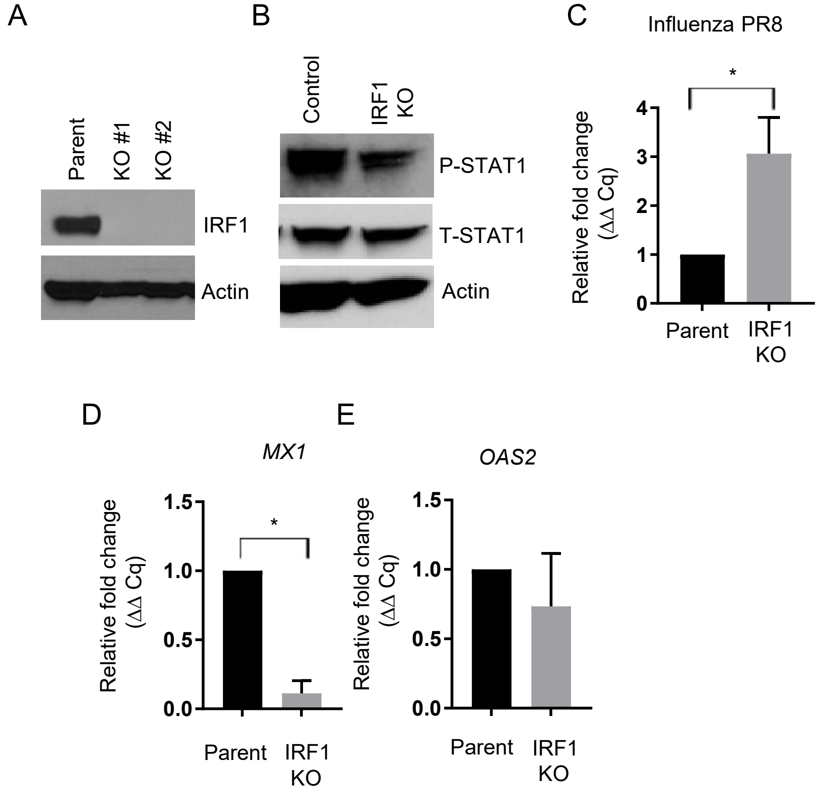

Supplementary Figure 8

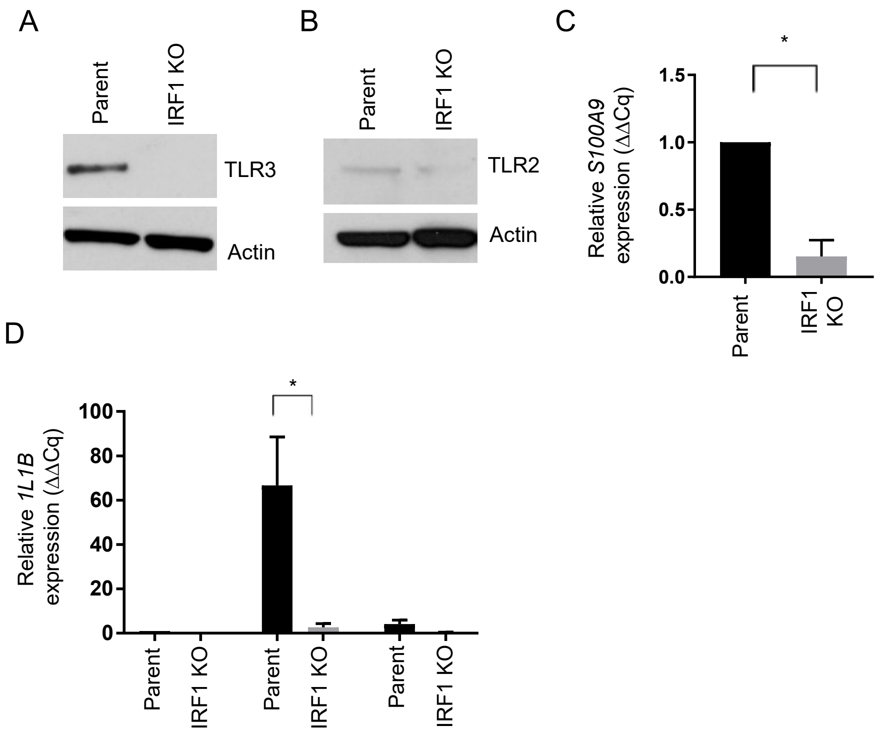

Supplementary Figure 9

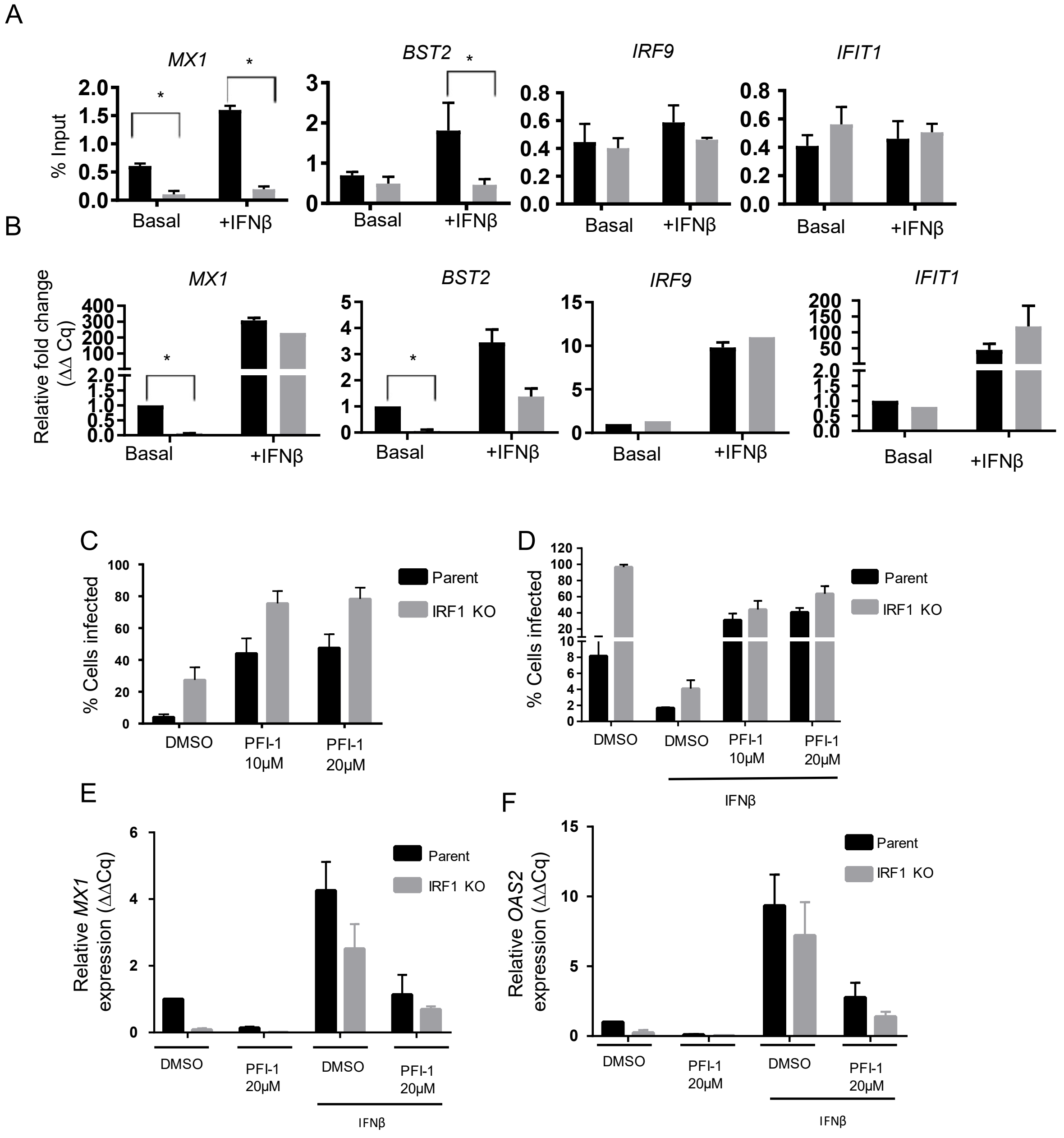

Supplementary Figure 1: Validation of IRF1 protein expression: (A) Parent BEAS-2B cells were treated with IFNβ (100ng/ml) for 2h and localization of IRF1 was examined using confocal microscopy. (B) Parent and two putative IRF1 KO BEAS-2B cell lines were treated with IFNβ (100ng/ml) for 2h and IRF1 protein expression was examined by western-blot.

Supplementary Figure 2: IRF1 protects cells against influenza and VSV infections. (A-D) Parent and IRF1 KO cells were infected with different strains of influenza virus and influenza HA gene expression was examined by RT-qPCR. Relative fold change ($2^{-\Delta\Delta Cq}$) is shown. Data represent mean ± SD. (E) Parent and a second IRF1 KO clonal cell population (IRF1 KO #2) were infected with VSV at 0.01 MOI for 22h. VSV infection was examined by flow cytometry. Percent cells infected is shown. * Statistically significant.

Supplementary Figure 3: VSV infection induces IFN transcript expression. (A) Constitutive *IRF3* gene expression was examined by RT-qPCR in parent and IRF1 KO cells. Relative fold change ($2^{-\Delta\Delta Cq}$) is shown. (B-C) Parent BEAS-2B and IRF1 KO cells were infected with VSV at 0.01 MOI for 6h. Expression of VSV-N mRNA (B) and IFNβ, IFNλ1 or IFNλ2 transcripts (C) were examined by RT-qPCR. Data represent mean from two independent experiments.

Supplementary Figure 4: Blockade of IFN-dependent gene expression. (A-B) Parent and IRF1 KO BEAS-2B cells were either left untreated or treated with B18R or Y136 proteins 24h before treating with IFNβ at 0.2ng/ml (A) or IFNλ1 at 5ng/ml (B). IFN-inducible gene expression was examined after 24h of IFN treatments using RT-qPCR. Data represent mean from two independent experiments. (C-D) Parent and IRF1 KO BEAS-2B cells were either treated with vehicle control or with ruxolitinib (Rux) at 10μM 24h before IFN treatment and then treated with IFNβ at 0.2ng/ml (C) and IFNλ1 at 5ng/ml (D) for 24h. IFN-inducible gene expression was examined by RT-qPCR. Data represent mean±SD from three experiments. (E-F) Parent and IRF1 KO BEAS-2B cells were

either treated with vehicle control or with AZD1480 at 5µM 24h before IFN treatment and then treated with IFNβ at 0.2ng/ml (E) and IFNλ1 at 5ng/ml (F) for 24h. IFN-inducible gene expression was examined by RT-qPCR. Data represent mean from two independent experiments.

Supplementary Figure 5: Comparison of gene expression in parent and IRF1 knockout cells upon IFNβ and IFNλ1 treatments. (A-B) A scatter plot of RNA-Seq read counts for all representative genes from the Cufflinks analysis in parent untreated and IFNβ treated cells (A) and IRF1 KO untreated and IFNβ treated cells (B). The values are expressed as log2 FPKM. Specific subsets of transcripts that are either upregulated or down regulated (2 Fold and FDR <0.01) due to IFNβ treatment are shown in red. Some of the important IFNβ ISGs are highlighted as text. (C) A volcano plot of RNA-Seq read counts for all representative genes from the Cufflinks analysis in parent cells treated with IFNβ compared to IRF1 KO cells treated with IFNβ. Specific subsets of transcripts that are either upregulated or down regulated (2 Fold and FDR <0.01) due to IFNβ treatment are shown in red. (D-E) A scatter plot of RNA-Seq read counts for all representative genes from the Cufflinks analysis in parent untreated and IFNλ1 treated cells (D) and IRF1 KO untreated and IFNλ1 treated cells (E) expressed as log2 FPKM. Specific subsets of transcripts that are either upregulated or down regulated (2 Fold and FDR <0.01) due to IFN λ1 treatment are shown in blue. Some of the important IFNλ1 dependent ISGs are highlighted as text. (F) A volcano plot of RNA-Seq read counts for all representative genes from the Cufflinks analysis in parent cells treated with IFNλ1 compared to IRF1 KO cells treated with IFNλ1. Specific subsets of transcripts that are either upregulated or down regulated (2 Fold and FDR <0.01) due to IFNλ1 treatment are shown in blue.

Supplementary Figure 6: Pathway analysis for IRF1 dependent genes. (A-B) Heatmap analysis of top 30 IRF1-dependent genes, either down regulated (A) or upregulated (B) is shown. (C)

Genes that showed reduced expression in IRF1 KO cells were examined for enriched pathways using Ingenuity Pathway Analysis. Pathways that showed significant enrichment are shown. (D) Interaction network analysis of genes that showed reduced expression in IRF1 KO cells. Four networks involving immune related genes are shown.

Supplementary Figure 7: IRF1 regulates antiviral response in A549 cells. (A) Parent and two putative IRF1 KO A549 cell lines were treated with IFNβ (100ng/ml) for 2h and IRF1 protein expression was examined by western-blot. (B) IRF1 KO and parent A549 cells were transfected with poly I:C and cell lysates were immunoblotted for STAT1 phosphorylation (Y701). (C) Parent and IRF1 KO A549 cells were infected with PR8 strain of influenza virus and influenza M gene expression was examined by RT-qPCR. Relative fold change ($2^{-\Delta\Delta Cq}$) is shown. Data represent mean ± SD from three independent experiments. (D-E) Basal expression for *MX1* and *OAS2* in A549 parent and IRF1KO cells is shown. Relative fold change ($2^{-\Delta\Delta Cq}$) is shown. * Statistically significant.

Supplementary Figure 8: IRF1 regulates TLR3 and TLR2 signaling. (A-B) Constitutive level of TLR3 and TLR2 protein expression was examined by western-blot. (C) Constitutive *S100A9* gene expression was examined by RT-qPCR in parent and IRF1 KO cells. Relative fold change ($2^{-\Delta\Delta Cq}$) is shown. (D) Parent and IRF1 KO cells were treated with the TLR2 agonist Pam2CSK4 and activation of NF-kB pathway was examined by RT-qPCR analysis of *IL1B*. Relative fold change ($2^{-\Delta\Delta Cq}$) is shown. * Statistically significant.

Supplementary Figure 9: IRF1 regulates promoter activation. (A) Parent and IRF1-KO cells were treated with IFNβ or left untreated. Chromatin immunoprecipitation was done using IRF1 antibody and the promoter regions harboring potential IRF1 binding sites of IRF1 dependent genes (MX1 and BST2) and IRF1 independent genes (IRF9 and IFIT1) were analyzed. Percent input (mean ±

standard deviation of two biological replicate experiments and three technical replicate PCRs) is expressed as relative enrichment over input (right axis) at steady state and upon IFNβ treatment. (B) In parallel experiments, IFNβ inducible gene expression was analyzed. Relative fold change ($2^{-\Delta\Delta Cq}$) is shown for *MX1*, *BST2*, *IRF9* and *IFIT1*. (C) Parent and IRF1 KO BEAS-2B cells were either treated with DMSO or PFI-1 for 6h before infection with VSV (MOI=0.01). Cells were harvested 20 hpi for analysis of GFP by flow cytometry. Data represent mean± SEM from three independent experiments. (D) Parent and IRF1 KO BEAS-2B cells were either treated with DMSO or PFI-1 for 2h and then treated with IFNβ (0.1ng/ml) for 4 h, after which they were infected with VSV (MOI=0.01) for 20h. IFNβ and PFI-1 concentrations were maintained throughout the infection. Percent cells infected was examined by flow cytometry. (E-F) Parent or IRF1 KO cells were either treated with DMSO or PFI-1 for 2h and then with IFNβ (0.1 ng/mL) for 20h. *OAS2* and *MX1* expression was examined by RT-qPCR at 24h after IFN treatment. Relative fold change ($2^{-\Delta\Delta Cq}$) is shown. Data represent mean± SEM from three independent experiments. * Statistically significant.
